# Substrate specificity of a ketosynthase domain involved in bacillaene biosynthesis

**DOI:** 10.3762/bjoc.20.67

**Published:** 2024-04-05

**Authors:** Zhiyong Yin, Jeroen S Dickschat

**Affiliations:** 1 Kekulé-Institute of Organic Chemistry and Biochemistry, University of Bonn, Gerhard-Domagk-Straße 1, 53121 Bonn, Germany

**Keywords:** bacillaene, biosynthesis, enzyme mechanisms, isotopes, *trans*-AT polyketide synthases

## Abstract

An isotopic labelling method was developed to investigate substrate binding by ketosynthases, exemplified by the second ketosynthase of the polyketide synthase BaeJ involved in bacillaene biosynthesis (BaeJ-KS2). For this purpose, both enantiomers of a ^13^C-labelled *N*-acetylcysteamine thioester (SNAC ester) surrogate of the proposed natural intermediate of BaeJ-KS2 were synthesised, including an enzymatic step with glutamate decarboxylase, and incubated with BaeJ-KS2. Substrate binding was demonstrated through ^13^C NMR analysis of the products against the background of various control experiments.

## Introduction

Polyketides are a large class of natural products which often exhibit potent biological activities for their application in medicine, e.g., as antibiotics or immunosuppressants [1]. Despite their high structural variability all compounds from this class are commonly made through the action of polyketide synthases (PKS). The type I of these enzymes are megasynthases composed of several catalytically active domains that can either act iteratively with the same set of domains catalysing the incorporation of several extender units into a growing polyketide chain, or non-iteratively with one set of domains acting only for the incorporation of one extender unit [2–3]. Although enzyme domains with various specialised catalytic functions can be found as integral part of polyketide synthases, three domain types are fundamental to their biosynthesis, resembling the same logic as observed for fatty acid biosynthesis: the acyl transferases (AT) for loading of the starter or extender units, the acyl carrier proteins (ACP) for anchoring the growing polyketide chain, and the ketosynthases (KS) for merging of the next extender unit with the existing chain by a decarboxylative Claisen condensation [2,4]. Today a high understanding of polyketide biosynthesis has been reached, including a detailed knowledge of the extender unit selection and the stereochemical implications that are predictable from amino acid sequences [5–6].

**Scheme 1:**
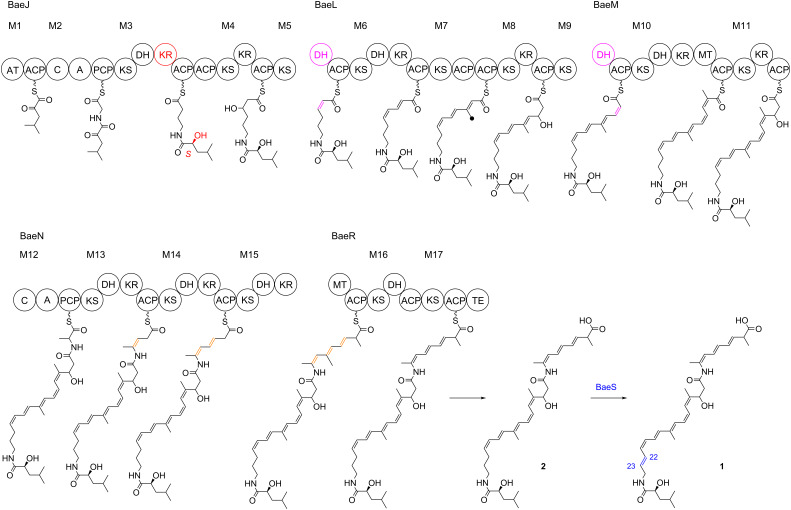
Biosynthetic model for bacillaene (**1**). M1–M17 indicate modules 1–17. A = adenylation domain, ACP = acyl carrier protein, AT = acyltransferase, C = condensation domain, DH = dehydratase, KR = ketoreductase, KS = ketosynthase, MT = methyltransferase, PCP = peptidyl carrier protein, TE = thioesterase. The black dot indicates a methyl branch introduced by the β-branching cassette.

The polyene antibiotic bacillaene was first isolated from *Bacillus subtilis* [7]. This soil-dwelling organism shares the same habitat with the predator *Myxococcus xanthus* that feeds on other bacteria including *B. subtilis*, and bacillaene is the primary factor conferring *B. subtilis* cells resistance to predation by *M. xanthus* [8]. The identification of its biosynthetic gene cluster (*bae*) revealed that the compound is made through a *trans*-AT polyketide synthase–non-ribosomal peptide synthase (PKS-NRPS) hybrid [9–10]. Instead of using the classical domain organisation KS-AT-ACP with AT domains integrated into the PKS, *trans*-AT PKSs utilize discrete ATs that are not an integral part of the PKS, but rather cooperate with the PKS “*in trans*” [11–12]. Notably, in *B. subtilis* the giant bacillaene biosynthesis machinery forms an organelle-like complex that can be observed through cryoelectron microscopy [13]. The structure elucidation of “bacillaene” through extensive NMR spectroscopic methods revealed the presence of two major compounds, bacillaene (**1**) and dihydrobacillaene (**2**) (Scheme 1), and allowed for a detailed biosynthetic model [14]. As is typical for *trans*-AT systems, the bacillaene PKS-NRPS contains several irregular features such as split modules and duplicate ACP domains. Because of the absence of a ketoreductase (KR) domain in module 1, the starter unit was initially suggested to be α-hydroxyisocaproate [14], but later it was shown that the KR of module 3 acts twice, in the reduction of the β-ketoacyl intermediate of the elongation step of module 3 and in the reduction of the α-ketoisocaproate starter unit with introduction of an *S* configured stereocentre (highlighted in red in Scheme 1) [15]. The domain organisation of module 3 containing no enoylreductase (ER) domain furthermore suggests the formation of an α,β-unsaturated intermediate, and not a full reduction at this stage, in agreement with the presence of a double bond between C22 and C23 in **1**. A contrasting picture was obtained through deletion of the TE domain that resulted in off-loading of all premature intermediates from the PKS [16], possibly catalysed by a proofreading AT-like enzyme encoded in the *bae* cluster [17]. These intermediates consistently showed masses two Da higher than expected, in agreement with a fully saturated intermediate at module 3 [16]. Subsequent investigations demonstrated that the C22=C23 double bond in **1** is incorporated in a post-PKS step through the action of BaeS (highlighted in blue) [18].

Further interesting features are the presence of a β-branching cassette for the installation of a β-methyl group at the stage of the module 7 intermediate (highlighted by the black dot) [14,16], and split modules between BaeJ and BaeL (module 5), as well as between BaeL and BaeM (module 9). These modules are suggested not to catalyse elongations, but the KS domains of modules 5 and 9 may only act in the translocation of the intermediate from the ACP of the previous module to the ACP of modules 5 and 9, respectively. Modules 5 and 9 then only catalyse the dehydration of the alcohol functions installed by the preceding modules (highlighted in purple) [14,16]. Furthermore, the structures of **1** and **2** show a shifted triene portion that is not in conjugation with the carboxylic acid function. NMR studies of off-loaded intermediates with the TE deletion mutant revealed that these double bond shifts are introduced during the elongation steps of modules 13–15, and not after complete assembly of the PKS backbone [19].

A phylogenetic analysis of KS domains from *trans*-AT PKSs revealed that these domains group together, if the structures of their processed substrates are similar, allowing for a prediction of the substrate specificity of such KS domains [20]. Especially the KS domains of a PKS module downstream of an NRPS module need to be able to process unconventional NRPS-derived intermediates [21]. Despite the above mentioned predictability of KS domain substrate specificities, functional testing of KS domains is still important. Previous approaches to determine KS domain specificities have involved mass spectrometry (MS)-based methods [22–23], MS analysis of trypsin-digested proteins [24], and radiochemical assays [25]. Here, we report on a new method using ^13^C-labelled substrate surrogates in conjunction with ^13^C NMR to investigate substrate acceptance by BaeJ-KS2 from module 4 of the bacillaene PKS.

## Results and Discussion

### Synthesis of *N*-acetylcysteamine thioesters

**Scheme 2:**
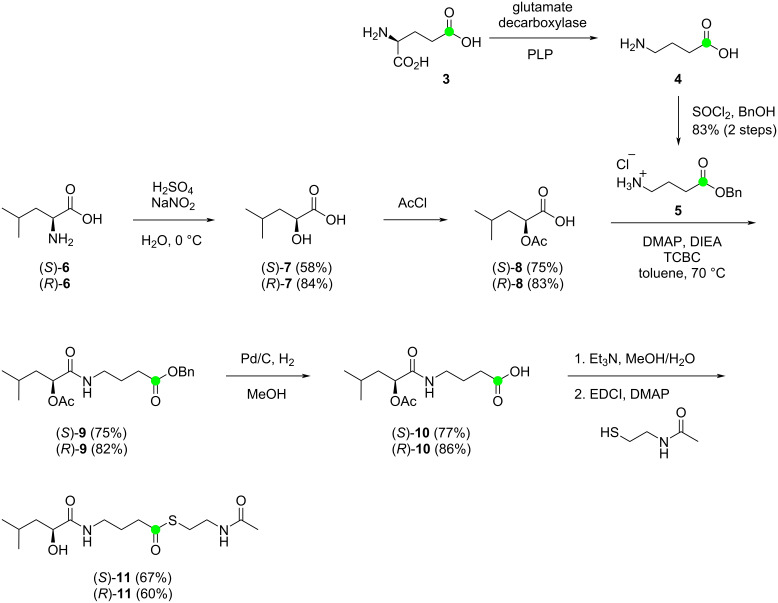
Synthesis of the BaeJ-KS2 substrate surrogates (*S*)-**11** and (*R*)-**11**. Green dots represent ^13^C-labelled carbons.

Previous studies have shown that *N*-acetylcysteamine thioesters (SNAC esters) can be uploaded to KS domains [22–25]. Therefore, to investigate the function of the KS domain BaeJ-KS2 the synthesis of ^13^C-labelled (*S*)-**11** as a mimic of the intermediate bound to the ACP of module 3 was performed. It was planned to introduce the ^13^C-labelling from (5-^13^C)glutamate into the γ-aminobutyrate portion of (*S*)-**11**. For this purpose, the gene coding for the glutamate decarboxylase from *Escherichia coli* K12 (accession no. AAA23833) was cloned through homologous recombination in yeast into the expression vector pYE-Express [26]. Heterologous expression and purification of the recombinant enzyme (Figure S1, Supporting Information File 1) allowed for test incubations with unlabelled glutamic acid in the presence of the cofactor pyridoxal phosphate (PLP), showing the complete conversion of glutamic acid (**3**) into γ-aminobutyric acid (**4**) (Scheme 2). The crude product was used without purification for a Schotten–Baumann esterification with benzyl alcohol to obtain the unlabelled ester **5** with a quantitative yield over two steps. After having established this method, (5-^13^C)glutamic acid (**3**) was converted analogously into (1-^13^C)-**5**, unfortunately with a little lower, but still very good yield of 83%.

By employing Yamaguchi conditions, the ester **5** was coupled with the carboxylic acid (*S*)-**8**, derived from ʟ-leucine ((*S*)-**6**) via hydroxyacid (*S*)-**7**, to yield the amide (*S*)-**9**. Deprotection through catalytic hydrogenation to (*S*)-**10**, saponification of the acetate ester and Steglich esterification with *N*-acetylcysteamine gave access to the desired SNAC ester (*S*)-**11**, with a yield of 32% over 5 steps starting from ^13^C-labelled **3**. Analogous reactions were performed to obtain (*R*)-**11** from ᴅ-leucine ((*R*)-**6**) with a comparable overall yield of 35% from labelled **3**.

### BaeJ-KS2 activity assay

The substrate specificities of ketosynthases in *trans*-AT PKSs hold significant importance. Many KSs within *trans*-AT PKSs function as gatekeepers, facilitating the transfer of intermediates along the assembly line without participating in chain elongation. For instance, the final KS of the bacillaene PKS lacks condensation capability, but may serve to ensure proper double bond isomerisation in the late polyketide intermediates before they are passed on for their TE-mediated release from the PKS. Such gatekeeping roles have also been discussed for condensation-competent KSs [27] that may process the substrate for the next round of elongation only after installation of the correct functional groups through optional reductive loop or other modifying domains. Located in module 4, BaeJ-KS2 was in the focus of our investigation to assess its response to both enantiomers of the full-length substrate surrogate **11**.

**Figure 1:**
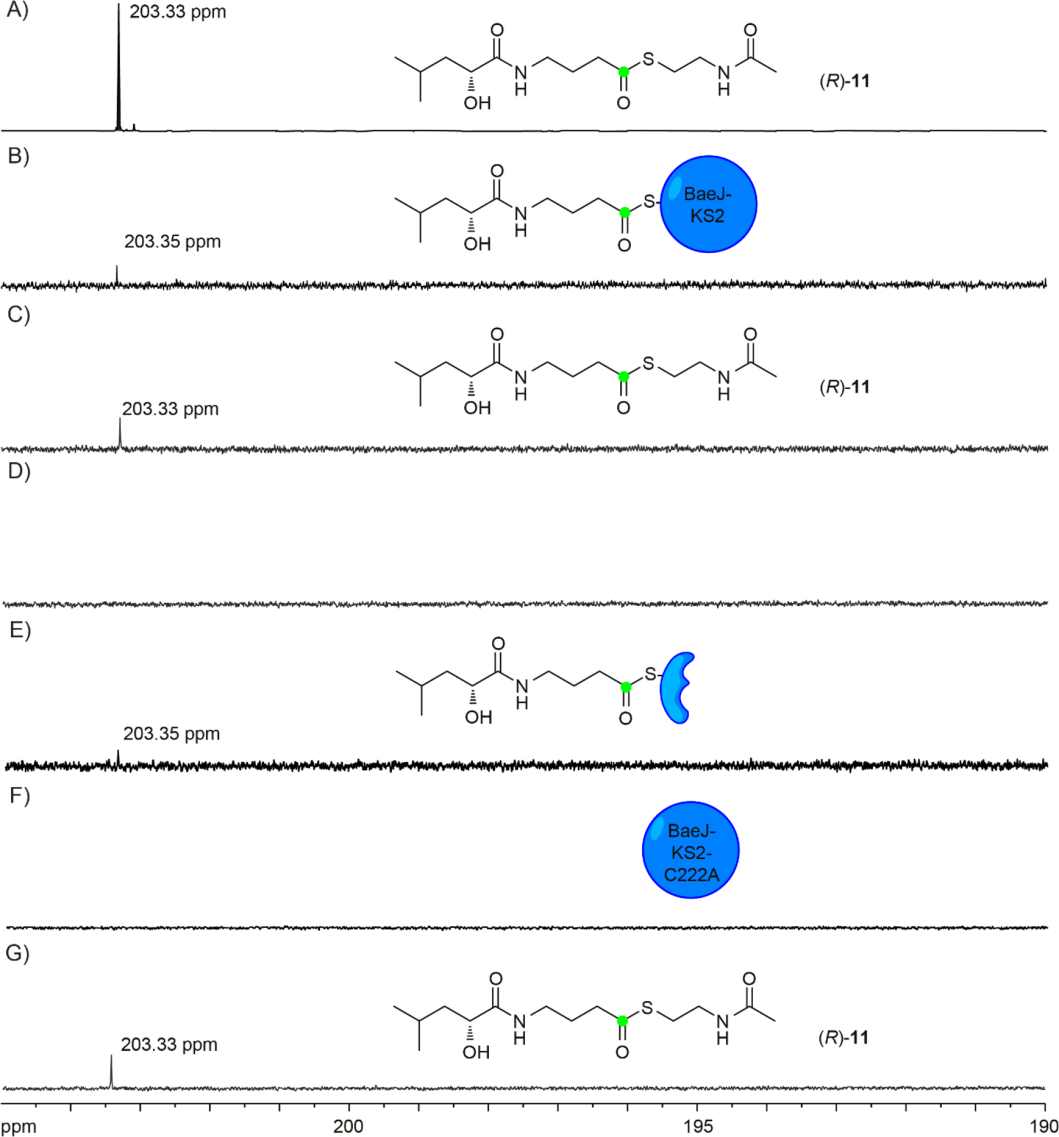
^13^C NMR spectra of (*R*)-**11** incubated with BaeJ-KS2 and BaeJ-KS2-C222A. A) Free **11** dissolved in incubation buffer; B) (*R*)-**11** bound to BaeJ-KS2 after incubation and buffer exchange (5 centrifugations); C) the filtrate obtained from the incubation of (*R*)-**11** BaeJ-KS2 containing free (*R*)-**11** (first centrifugation); D) the filtrate from the same experiment containing no (*R*)-**11** (fifth centrifugation); E) the filtrate obtained from the incubation of (*R*)-**11** with BaeJ-KS2 followed by buffer exchange and then digestion with proteinase K; F) (*R*)-**11** is not bound to BaeJ-KS2-C222A after incubation and buffer exchange (5 centrifugations); G) the filtrate obtained from the incubation of (*R*)-**11** BaeJ-KS2-C222A containing free (*R*)-**11** (first centrifugation). Green dots represent ^13^C-labelled carbons.

To investigate the function of BaeJ-KS2 the recombinant His-tagged enzyme was obtained through heterologous expression and purification by Ni^2+^-NTA affinity chromatography (Figure S1, Supporting Information File 1). After incubation of the purified enzyme with the ^13^C-labelled SNAC derivatives (*S*)-**11** or (*R*)-**11** for 30 minutes at 25 °C, the incubation buffer of the reaction mixture potentially containing the free substrate surrogates was exchanged through repeated centrifugation using an ultrafiltration centrifugal tube (3 kDa cut-off), followed by the addition of incubation buffer. In total, through this method five successive ≈10:1 dilutions were performed to eliminate any unreacted free **11** in the reaction mixture. The resulting protein preparations were subsequently analysed by ^13^C NMR spectroscopy. While the signal for the thioester carbonyl group of free **11** dissolved in incubation buffer was observed at δ = 203.33 ppm (Figure 1A), for both samples obtained from the incubations with (*S*)-**11** and (*R*)-**11** signals were detected with a small, but reproducible difference of the chemical shift at δ = 203.35 ppm (Figure 1B), likely representing enzyme bound **11**. Furthermore, the filtrates obtained from the first and the fifth centrifugation were analysed by ^13^C NMR, showing the presence of free **11** after the first centrifugation step (Figure 1C), but not after the last round of centrifugation (Figure 1D).

Protein binding of the substrate surrogates **11** was confirmed through digestion of BaeJ-KS2 using protease K after buffer exchange. The digested sample was subsequently subjected to another round of centrifugation using an ultrafiltration centrifugal tube, resulting in the detection of a signal for a thioester carbonyl group in the filtrate for both samples derived from (*S*)- and (*R*)-**11** at δ = 203.35 ppm (Figure 1E). This observation supports binding of both substrate surrogates to BaeJ-KS2, but it is unclear from these experiments, if **11** is bound covalently to the protein or through non-covalent interactions.

To gain further evidence for the covalent binding of **11** to BaeJ-KS2, the highly conserved Cys residue involved in substrate attachment [28–29] was exchanged through site-directed mutagenesis, resulting in the BaeJ-KS2-C222A enzyme variant. After heterologous expression and protein purification (Figure S1, Supporting Information File 1), the same protocol for the incubation with (*S*)-**11** and (*R*)-**11** followed by exchange of the incubation buffer as described above was applied. No signal corresponding to a thioester carbonyl group was detected in the protein preparations (Figure 1F), while signals at δ = 203.33 ppm for free **11** were observed in the filtrates obtained after the first step (Figure 1G).

## Conclusion

Taken together, we have established a new method based on stable isotope (^13^C) labelling to investigate the KS domain substrate specificity that makes use of simple ^13^C NMR analysis of protein preparations obtained by a buffer exchange after enzyme incubations with substrate surrogates (SNAC esters), and its application to investigate BaeJ-KS2 from the bacillaene PKS. While this enzyme has been investigated for its substrate scope before [27], the present study is the first that investigates the acceptance of a substrate surrogate representing the natural module 3 intermediate. The obtained results confirm the structure of this intermediate as *N*-((*S*)-α-hydroxyisocaproyl)-γ-aminobutanoyl-S-ACP and also demonstrate that BaeJ-KS2 is not sensitive towards an inversion of the configuration in the α-hydroxyisocaproate moiety. This finding is in agreement with the phylogenetic analysis of KS domains from *trans*-AT PKSs, showing that the amino acid sequences of these KSs correlate with the structures of the processed PKS intermediates up to the β-carbon [20].

## Supporting Information

File 1 Experimental part and NMR spectra.

## Data Availability

All data that supports the findings of this study is available in the published article and/or the supporting information to this article.
