# Supplementary material for: Substrate specificity of a ketosynthase domain involved in bacillaene biosynthesis
Source: Beilstein J Org Chem. 2024 Apr 5;20:734–40. doi: 10.3762/bjoc.20.67 (PMC10999986; doi:10.3762/bjoc.20.67)
Supplement: File 1 — Experimental part and NMR spectra. [file Beilstein_J_Org_Chem-20-734-s001.pdf]

BEILSTEIN JOURNAL OF ORGANIC CHEMISTRY

Supporting Information

for

Substrate specificity of a ketosynthase domain involved in bacillaene biosynthesis

Zhiyong Yin and Jeroen S. Dickschat

Beilstein J. Org. Chem. **2024,** *20,* 734–740. doi:10.3762/bjoc.20.67

Experimental part and NMR spectra

License and Terms: This is a supporting information file under the terms of the Creative Commons Attribution License (https://creativecommons.org/licenses/by/4.0). Please note that the reuse, redistribution and reproduction in particular requires that the author(s) and source are credited and that individual graphics may be subject to special legal provisions.
The license is subject to the *Beilstein Journal of Organic Chemistry* terms and conditions: (https://www.beilstein-journals.org/bjoc/terms)

Strains and culture conditions

Bacillus amyloliquefaciens FZB42 was grown in GYM medium (5.0 g peptone, 4.0 g meat extract, 10.0 mg $MnSO_4$, 1 L water, pH 7.0) at 30 °C. *Escherichia coli* K12 (DSM 18039) and *E. coli* BL21 (DE3) were grown in LB medium at 37 °C. *Saccharomyces cerevisiae* was grown in YPAD medium (20 g glucose, 10 g yeast extract, 20 g peptone, 40 mg adenine sulfate, 1 L water) at 30 °C.

Gene cloning

The gene sequences encoding the glutamate decarboxylase from *E. coli* K12 (accession number AAA23833) [1], BaeJ-KS2 from *B. amyloliquefaciens* FZB42 (spanning the amino acid residues 3527–3937 of BaeJ, accession number AJ634060) [2], and BaeJ-KS2-C222A were cloned into the pYE-Express expression vector [3] through homologous recombination in yeast. For this purpose, the wildtype genes were amplified from gDNA using the short primers listed in Table S1, followed by a second PCR using the PCR product as a template and the long primers listed in Table S1 containing homology arms with homologous sequences to the end sequences of the linearised pYE-Express vector (EcoRI and HindIII digestion). The mutated gene coding for BaeJ-KS2-C222A was obtained through mutational PCR, followed by analogous attachment of the homology arms through PCR. Q5 High-fidelity DNA polymerase (New England Biolabs, Ipswich, MA, USA) was used for all PCRs.

The glutamate decarboxylase gene sequence was amplified from gDNA from *E. coli* K12 with primers YZ01_Fw and YZ02_Rv. PCR conditions were: initial denaturation at 98 °C, 1 min; 3-step cycle: 98 °C, 10 s; 58.1 °C, 30 s; 72 °C, 45 s; repeated 35 times; final elongation at 72 °C, 2 min. The obtained PCR products were elongated with homology arms by PCR using the primers YZ03_LFw and YZ04_LRv under the same PCR conditions.

The BaeJ-KS2 gene sequence was amplified from gDNA from *B. amyloliquefaciens* FZB42 using primers YZ019_Fw and YZ020_Rv. PCR conditions were: initial denaturation at 98 °C, 30 s; 3-step cycle: 98 °C, 10 s; 60.3 °C, 30 s; 72 °C, 62 s; repeated 35 times; final elongation at 72 °C, 2 min. The obtained PCR products were elongated with homology arms by PCR using the primers YZ021_LFw and YZ022_LRv under the same PCR conditions (Table S1).

The coding sequence of BaeJ-KS2-C222A was obtained by site-directed mutation. Therefore, two overlapping gene fragments carrying the mutation in the overlapping

sequence were obtained by PCR using plasmid pYE-BaeJ as a template (a definition for this plasmid is given below). The first fragment was ontained using primers YZ021_LFw and YZ115_Rv and the following temperature program: initial denaturation at 98 °C for 30 s; 3-step cycle: 98 °C for 10 s, 60.3 °C for 30 s, 72 °C for 18 s; repeated 35 times; final elongation at 72 °C for 2 min. The second fragment was ontained using primers YZ114_Fw and YZ022_LRv and the following temperature program: initial denaturation at 98 °C for 30 s; 3-step cycle: 98 °C for 10 s, 60.3 °C for 30 s, 72 °C for 45 s; repeated 35 times; final elongation at 72 °C for 2 min. The two fragments were isolated through a plasmid purification kit (Wizard SV Gel and PCR Clean-Up System; Promega, Madison, Wisconsin, USA) and mixed for a third PCR using the following temperature program: initial denaturation at 98 °C for 30 s; 3-step cycle: 98 °C for 10 s, 60.3 °C for 30 s, 72 °C for 60 s; repeated 5 times; final elongation at 72 °C for 2 min. At this stage the long primers YZ021_LFw and YZ022_LRv were added for attachment of the homology arms. The PCR was then continued using the following temperature program: initial denaturation at 98 °C for 30 s; 3-step cycle: 98 °C for 10 s, 60.3 °C for 30 s, 72 °C for 60 s; repeated 35 times; final elongation at 72 °C for 2 min.

The PCR products together with the linearised pYE-Express shuttle vector (EcoRI and HindIII digestion) were used for a yeast homologous recombination by a standard protocol using PEG, LiOAc and salmon sperm DNA [3]. Cells were plated on YPAD medium and the cultures were grown at 28 °C for 2 days. Plasmid DNA was isolated using the Zymoprep™ Kit (Yeast Plasmid Miniprep) and then shuttled into *E. coli* electrocompetent cells by electroporation. These cells were grown overnight at 37 °C on LB agar plates (kanamycin 50 µg mL^{-1}). Single colonies were selected to inoculate LB medium (10 mL) with kanamycin (10 µL; 50 mg mL^{-1}), followed by culturing for 12 h and isolation of plasmid DNA. The sequences of the cloned genes were verified by DNA sequencing. The plasmids were named pYE-GD (*E. coli* K12 glutamate decarboxylase), pYE-BaeJ (*B. amyloliquefaciens* FZB42 BaeJ), and pYE-BaeJ-C222A (C222A enzyme variant of BaeJ).

Table S1. Primers used for gene cloning.

Primer	Sequence[a]
YZ001_Fw	ATGGACCAGAAGCTGTTAAC
YZ002_Rv	TCAGGTGTGTTTAAAGCTG
YZ003_LFw	GGCAGCCATATGGCTAGCATGACTGGTGGAATGGACCAGAAGCTGTTAAC
YZ004_LRv	TCTCAGTGGTGGTGGTGGTGGTGCTCGAGTTCAGGTGTGTTTAAAGCTG
YZ019_Fw	GCAAAAGAGCATCCGGGCCGTTT
YZ020_Rv	TCACCATTGTTTCGTCAGAATACGTTTCATCCG
YZ021_LFw	GGCAGCCATATGGCTAGCATGACTGGTGGAGCAAAAGAGCATCCGGGCCGTTT
YZ022_LRv	TCTCAGTGGTGGTGGTGGTGGTGCTCGAGTTCACCATTGTTTCGTCAGAATACGTTTCATCCG
YZ114_Fw	GAAACGGCA**GCA**TCAAGCTCGCTTG
YZ115_Rv	CGAGCTTGA**TGC**TGCCGTTTCAATCGG

[a] Homology arms for recombination in yeast fitting to the end sequences of the digested pYE-Express vector are underlined. Changed triplet codons in mutational primers are highlighted in bold.

Gene expression and protein purification

E. coli BL21 (DE3) cells harboring the expression plasmids (pYE-GD, pYE-BaeJ, or pYE-BaeJ-C222A, respectively) were used to inoculate a starter culture in LB medium (10 mL) supplied with kanamycin (50 µg/mL), which was grown with shaking at 37 °C overnight. The starter cultures were used to inoculate expression cultures (1/100 v/v) in LB medium (1 L) with kanamycin and the cells were grown with shaking at 37 °C until OD_{600} = 0.4–0.6 was reached. The cultures were cooled to 18 °C, before IPTG (0.4 mM final concentration) was added to induce expression. The cultures were shaken at the same temperature overnight and then centrifuged (3500*g*, 40 min, 4 °C). For the preparation of glutamate decarboxylase the medium was discarded and the cell pellet was resuspended in binding buffer (10 mL; 50 mM Na_2HPO_4, adjusted to pH 5.5 with a 3 M solution of citric acid in water, 4 °C) [4]. The cells were lysed by ultrasonication (10 × 1 min) under ice cooling. The cell debris was spun down (14600*g*, 10 min, 4 °C), the protein solution was filtered with disposable syringe filter (Macherey and Nagel GmbH & Co. KG), and loaded onto a Ni^{2+}-NTA affinity chromatography column (10 mL column volume; Ni-NTA superflow, Qiagen, Venlo, Netherlands). The column was washed with two column volumes of binding buffer (20 mL) to elute non-binding proteins, followed by desorption of the target protein from the stationary phase with two column volumes of elution buffer (20 mL; 20 mM Na_2HPO_4, 0.15 mM PLP,

500 mM imidazole, adjusted to pH 6.5 with a 3 M solution of citric acid in water, 4 °C) with fractionation. The fractions were analysed by SDS-PAGE and fractions containing pure protein were pooled (Figure S1) and used for incubation experiments. Finally, the elution buffer was replaced with incubation buffer (50 mM Na_2HPO_4, 0.15 mM PLP, adjusted to pH 5.0 with a 3 M solution of citric acid in water) through repeated centrifugation using an ultrafiltration centrifugal tube. The protein concentration was determined through Bradford assay [5] and adjusted to 6.5 mg/mL.

For the preparation of BaeJ-KS2 and BaeJ-KS2-C222A the medium was discarded and the cell pellet was resuspended in binding buffer (10 mL; 25 mM Tris, 0.5 M NaCl, 10% (v/v) glycerol, 10 mM imidazole, pH 7.6, 4 °C) [2]. The cells were lysed by ultrasonication (10 × 1 min). The cell debris was spun down (14600*g*, 10 min, 4 °C), the protein solution was filtered with disposable syringe filter, and loaded onto a Ni^{2+}-NTA affinity chromatography column (10 mL column volume; Ni-NTA superflow, Qiagen, Venlo, Netherlands). The column was washed with two volumes of binding buffer (20 mL) to elute non-binding proteins, followed by desorption of the target protein from the stationary phase with two column volumes of elution buffer (20 mL; 25 mM Tris, 0.5 M NaCl, 10% (v/v) glycerol, 10 mM imidazole, pH 7.6) with fractionation. The fractions were analysed by SDS-PAGE and fractions containing pure protein were pooled and used for incubation experiments (Figure S1). Finally, the elution buffer was replaced with incubation buffer (25 mM Tris, 0.5 M NaCl, 10% (v/v) glycerol, pH 7.6) through repeated centrifugation using an ultrafiltration centrifugal tube. The protein concentration was adjusted to 8.0 mg/mL.

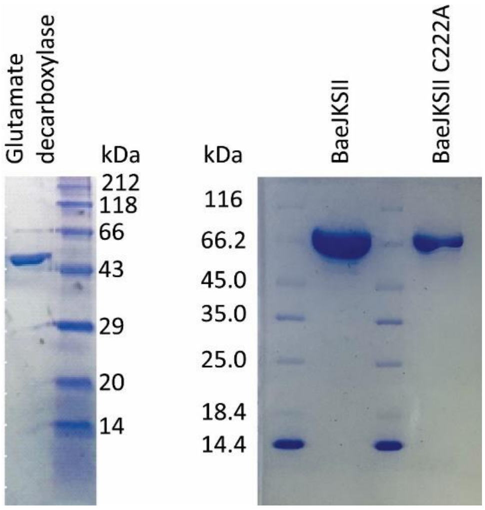

Figure S1. SDS-PAGE analysis of all recombinant enzymes used in this study. The theoretical molecular weights of the target proteins are 52.7 kDa (glutamate decarboxylase), 77.8 kDa (BaeJ KSII), 77.8 kDa (BaeJ KSII C222A).

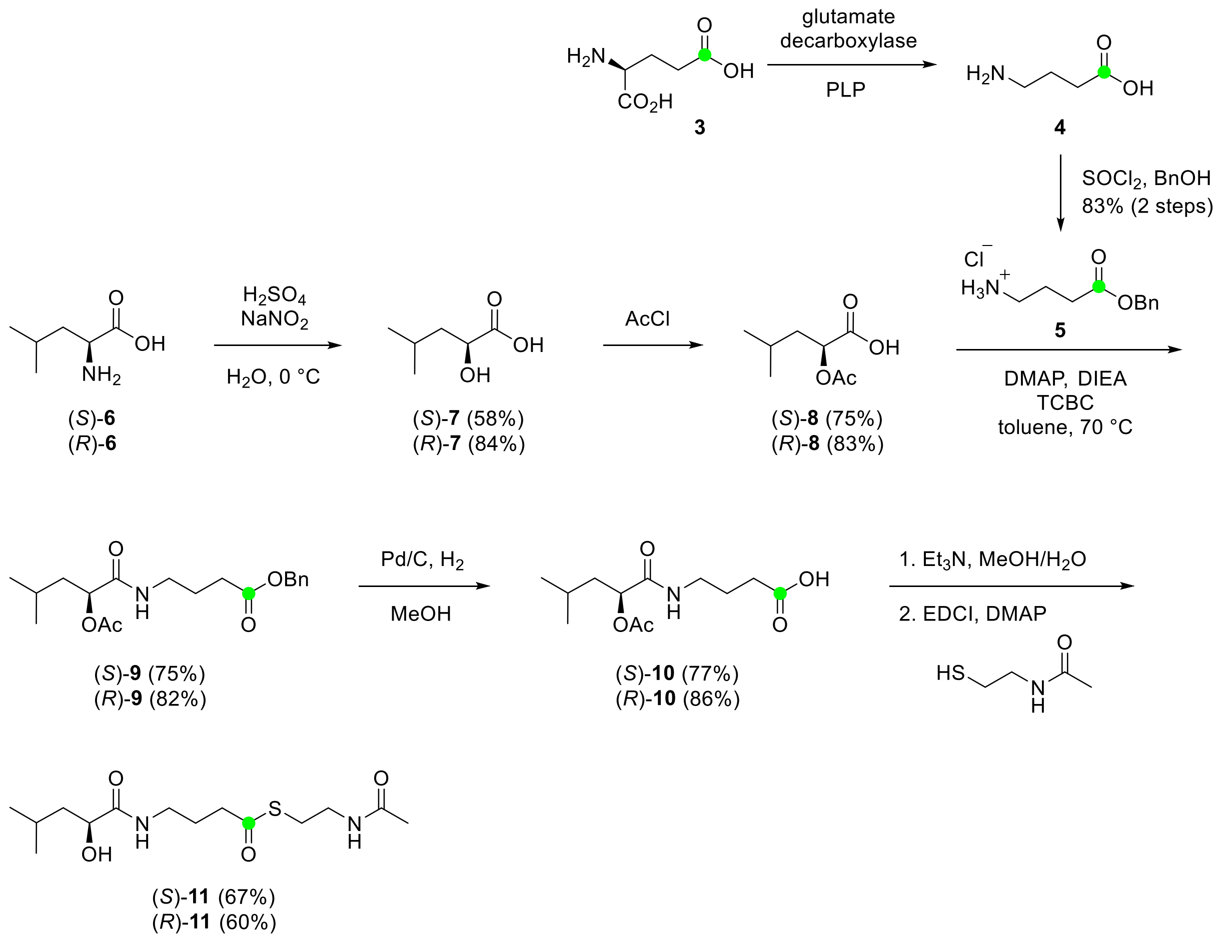

Scheme S1: Synthesis of the BaeJ-KS2 substrate surrogates (*S*)-**11** and (*R*)-**11**. Green dots represent ^{13}C-labelled carbons.

Enzymatic synthesis of (1-^{13}C)-γ-aminobutyric acid (4)

For the enzymatic synthesis of (1-^{13}C)-γ-aminobutyric acid (**4**), test reactions with unlabelled glutamic acid (**3**) in the presence of the cofactor pyridoxal phosphate (PLP) were conducted first. The reactions were performed in incubation buffer (50 mM Na_2HPO_4, 0.15 mM PLP, adjusted to pH 5.0 with a 3 M solution of citric acid in water). An enzyme preparation of glutamate decarboxylase (200 µL, 6.5 mg/mL) was added to a solution of glutamic acid (250 mg, 1.70 mmol) in incubation buffer (25 mL). The reaction mixture was incubated at 37 °C for 16 h. The mixture was concentrated to dryness under reduced pressure and directly analysed by NMR spectroscopy, showing the full conversion of glutamic acid into γ-aminobutyric acid (Figure S2). The crude product was used without purification for a Schotten–Baumann esterification with benzyl alcohol. Therefore, to a solution of unlabelled crude γ-aminobutanoic acid (**2**, 480 mg containing citric acid) in BnOH (6.89 g, 7.2 mL, 63.7 mmol), $SOCl_2$ (440 mg,

0.7 mL, 3.7 mmol) was added dropwise and the mixture was stirred overnight at room temperature. The reaction mixture was acidified by the addition of 1 N HCl (5 mL), followed by the extraction with diethyl ether (3 × 100 mL) to remove remaining BnOH. Unlabelled benzyl 4-aminobutanoate (**5**) (387 mg, 1.70 mmol, 100% over two steps) was obtained after filtration (Figure S3). Following this established method, (5-^{13}C)glutamic acid (**3**, 250 mg, 1.70 mmol) was converted analogously into (1-^{13}C)-**5** (324 mg, 1.41 mmol, 83% over two steps) (Figures S4–S7). ^{1}H NMR (500 MHz, MeOD): δ_H 7.38–7.29 (m, 5H), 5.14 (d, $^{3}J_{C,H}$ = 3.3 Hz, 2H), 2.98 (t, $^{3}J_{H,H}$ = 7.5 Hz, 2H), 2.53 (dt, $^{3}J_{H,H}$ = 7.3 Hz, $^{2}J_{C,H}$ = 7.3 Hz, 2H), 1.96 (m, 2H) ppm; ^{13}C NMR (126 MHz, C_6D_6): δ_C 173.8 ($^{13}C_q$), 137.5 (d, $^{3}J_{C,C}$ = 2.0 Hz, C_q), 129.6 (2xCH), 129.3 (2xCH), 129.3 (CH), 67.5 (d, $^{2}J_{C,C}$ = 2.6 Hz, CH_2), 40.1 (d, $^{3}J_{C,C}$ = 3.6 Hz, CH_2), 31.6 (d, $^{1}J_{C,C}$ = 57.8 Hz, CH_2), 23.8 (d, $^{2}J_{C,C}$ = 1.6 Hz, CH_2) ppm. NMR data are in agreement with those of unlabelled **5** [6].

Synthesis of (*S*)- and (*R*)-2-hydroxy-4-methylpentanoic acid ((*S*)-7 and (*R*)-7)

To a solution of concentrated H_2SO_4 (3.74 g) in H_2O (25 mL) was added leucine (*S*)-**6** (5.00 g, 38.1 mmol). $NaNO_2$ (5.26 g, 76.2 mmol) was dissolved in 40 mL H_2O and was added dropwise. The mixture was stirred for 2 d at room temperature and then extracted with EtOAc (3 × 60 mL). The combined organic phases were washed with brine and dried with $MgSO_4$. The solvent was removed under reduced pressure to obtain (*S*)-**7** as a colourless solid (2.92 g, 22.1 mmol, 58%) without purification. ^{1}H NMR (500 MHz, $CDCl_3$): δ_H 4.29 (dd, $^{3}J_{H,H}$ = 8.5 Hz, $^{3}J_{H,H}$ = 4.8 Hz, 1H), 1.90 (m, 1H), 1.64 (ddd, $^{2}J_{H,H}$ = 14.0 Hz, $^{3}J_{H,H}$ = 8.1 Hz, $^{3}J_{H,H}$ = 4.7 Hz, 1H), 1.61 (ddd, $^{2}J_{H,H}$ = 14.0 Hz, $^{3}J_{H,H}$ = 8.4 Hz, $^{3}J_{H,H}$ = 5.7, 1H), 1.15 (d, $^{3}J_{H,H}$ = 6.6 Hz, 6H) ppm; ^{13}C NMR (126 MHz, $CDCl_3$): δ_C 180.7 (C_q), 69.1 (CH), 43.3 (CH_2), 24.6 (CH), 23.3 (CH_3), 21.6 (CH_3) ppm. Optical rotation: $[\alpha]_D^{25}$ = –10.2 (*c* 0.40, MeOH), lit. $[\alpha]_D^{20}$ = –13.1 (*c* 1.0, EtOH) [7]. Following the same procedure as for (*S*)-**7**, (*R*)-**6** (5.00 g, 38.1 mmol) was converted into (*R*)-**7** that was obtained as a colourless solid (4.24 g, 32.1 mmol, 84%). Optical rotation: $[\alpha]_D^{25}$ = +12.1 (*c* 0.23, MeOH), lit. $[\alpha]_D^{20}$ = +10.7 (*c* 1.0, EtOH) [7].

Synthesis of (*S*)- and (*R*)-2-acetoxy-4-methylpentanoic acid ((*S*)-8 and (*R*)-8)

A mixture of (*S*)-**7** (800 mg, 6.05 mmol) in acetyl chloride (8 mL) was stirred at room temperature overnight. The mixture was then concentrated under reduced pressure and the residue was diluted with EtOAc and washed with brine to give the ester (*S*)-**8**

(785 mg, 4.51 mmol, 75%) as a colourless oil that was used for the next step without purification. ^{1}H NMR (500 MHz, $CDCl_3$): δ_H 10.24 (br s, COOH), 5.04 (dd, $^{3}J_{H,H}$ = 9.6 Hz, $^{3}J_{H,H}$ = 4.0 Hz, 1H), 2.13 (s, 3H), 1.86–1.75 (m, 2H), 1.71–1.64 (m, 1H), 0.97 (d, $^{3}J_{H,H}$ = 6.4 Hz, 3H), 0.93 (d, $^{3}J_{H,H}$ = 6.4 Hz, 3H) ppm; ^{13}C NMR (126 MHz, $CDCl_3$): δ_C 176.9 (C_q), 170.9 (C_q), 70.7 (CH), 38.7 (CH_2), 24.8 (CH), 23.1 (CH_3), 21.6 (CH_3), 20.7 (CH_3) ppm. Optical rotation: $[\alpha]_D^{25}$ = –20.9 (*c* 0.33, MeOH), lit. $[\alpha]_D^{20}$ = –25.6 (*c* 6, CH_2Cl_2) [8].

Following the same procedure as for (*R*)-**8**, (*R*)-**7** (1.00 g, 7.57 mmol) was converted into (*R*)-**8** that was obtained as a colourless solid (1.10 g, 6.31 mmol, 83%). Optical rotation: $[\alpha]_D^{25}$ = +22.1 (*c* 0.30, MeOH).

Synthesis of benzyl (*S*)- and (*R*)-4-(2-acetoxy-4-methylpentanamido)-(1-^{13}C)butanoate ((*S*)-9 and (*R*)-9)

A solution of (*S*)-**8** (108 mg, 0.62 mmol), *N*,*N*-diisopropylethylamine (DIEA, 126 mg, 171 µL, 0.98 mmol) and 2,4,6-trichlorobenzoyl chloride (TCBC, 193 mg, 124 µL, 0.79 mmol) in dry toluene (7 mL) was stirred for 3 hours under argon. A solution of **5** (100 mg, 0.52 mmol) and 4-dimethylaminopyridine (DMAP, 30 mg, 0.25 mmol) in toluene (1 mL) was slowly added dropwise. The mixture was stirred at 70 °C overnight. After the addition of ethyl acetate (50 mL), the organic layer was washed with sat. NH_4Cl (10 mL), $NaHCO_3$ (10 mL) and brine (10 mL). The organic layer was dried with $MgSO_4$ and concentrated under reduced pressure. The residue was purified by column chromatography on silica gel (cyclohexane/EtOAc, 2:1) to give (*S*)-**9** (114 mg, 0.33 mmol, 75%) as a colourless solid. ^{1}H NMR (500 MHz, $CDCl_3$): δ_H 7.39–7.31 (m, 5H), 6.35 (br s, NH), 5.18 (dd, $^{3}J_{H,H}$ = 7.3 Hz, $^{3}J_{H,H}$ = 5.7 Hz, 1H), 5.14 (dd, $^{2}J_{H,H}$ = 12.4 Hz, $^{3}J_{C,H}$ = 3.1 Hz, 1H), 5.11 (dd, $^{2}J_{H,H}$ = 12.4 Hz, $^{3}J_{C,H}$ = 3.2 Hz, 1H), 3.31 (dt, $^{3}J_{H,H}$ = 6.4, $^{3}J_{H,H}$ = 6.4, 2H), 2.45 (dddd, $^{2}J_{H,H}$ = 16.7 Hz, $^{2}J_{C,H}$ = 7.0 Hz, $^{3}J_{H,H}$ = 7.0 Hz, $^{3}J_{H,H}$ = 7.0 Hz, 1H), 2.41 (dddd, $^{2}J_{H,H}$ = 16.7 Hz, $^{2}J_{C,H}$ = 7.0 Hz, $^{3}J_{H,H}$ = 7.0 Hz, $^{3}J_{H,H}$ = 7.0 Hz, 1H), 2.15 (s, 3H), 1.93–1.81 (m, 2H), 1.74–1.62 (m, 3H), 0.94 (d, $^{3}J_{H,H}$ = 6.4 Hz, 3H), 0.92 (d, $^{3}J_{H,H}$ = 6.4 Hz, 3H) ppm; ^{13}C NMR (126 MHz, $CDCl_3$): δ_C 173.6 ($^{13}C_q$), 170.6 (C_q), 170.1 (C_q), 135.9 (d, $^{3}J_{C,C}$ = 2.2 Hz, C_q), 128.8 (2xCH), 128.5 (CH), 128.4 (2xCH), 72.9 (CH), 66.7 (d, $^{2}J_{C,C}$ = 2.6 Hz, CH_2), 41.0 (CH_2), 39.0 (d, $^{3}J_{C,C}$ = 3.0 Hz, CH_2), 31.9 (d, $^{1}J_{C,C}$ = 57.5 Hz, CH_2), 24.7 (CH), 24.4 (d, $^{2}J_{C,C}$ = 3.0 Hz, CH_2), 23.3 (CH_3), 21.9 (CH_3), 21.1 (CH_3) ppm. HRMS (APCI): $[M+H]^+$ calculated for $C_{18}{}^{13}CH_{27}NO_5H^+$ *m*/*z* 351.1996; found *m*/*z* 351.1991. Optical rotation: $[\alpha]_D^{25}$ = –8.2 (*c* 0.11, MeOH).

Following the same procedure as for (*S*)-**9**, (*R*)-**8** (100 mg, 0.62 mmol) was converted into (*R*)-**9** that was obtained as a colourless solid (124 mg, 0.36 mmol, 82%). Spectroscopic data were identical to those of (*S*)-**9**. Optical rotation: $[\alpha]_D^{25}$ = +9.1 (*c* 0.25, MeOH).

Synthesis of (*S*)- and (*R*)-4-(2-acetoxy-4-methylpentanamido)-(1-^{13}C)butanoic acid ((*S*)-10 and (*R*)-10

A mixture of (*S*)-**9** (40 mg, 0.11 mmol) and Pd/C (17 mg, 5% Pd) in CH_3OH (4 mL) was stirred in a H_2 atmosphere (10 bar) for 1 h. At the end of the reaction the catalyst was removed by filtration and the solvents were evaporated to obtain the product (*S*)-**10** (23 mg, 0.09 mmol, 77%) without purification. ^{1}H NMR (500 MHz, $CDCl_3$): δ_H 6.29 (br s, NH), 5.18 (dd, $^3J_{H,H}$ = 8.1 Hz, $^3J_{H,H}$ = 4.9 Hz, 1H), 3.42–3.29 (m, 2H), 2.44–2.37 (m, 1H), 2.16 (s, 3H), 1.91–1.83 (m, 2H), 1.75–1.63 (m, 3H), 0.94 (d, $^3J_{H,H}$ = 6.4 Hz, 3H), 0.92 (d, $^3J_{H,H}$ = 6.4 Hz, 3H) ppm; ^{13}C NMR (126 MHz, $CDCl_3$): δ_C 177.1 ($^{13}C_q$), 171.0 (C_q), 170.3 (C_q), 73.0 (CH), 41.0 (CH_2), 38.8 (d, $^3J_{C,C}$ = 2.7 Hz, CH_2), 31.4 (br d, $^1J_{C,C}$ = 56 Hz, CH_2), 24.7 (CH), 24.6 (CH_2), 23.2 (CH_3), 21.9 (CH_3), 21.1 (CH_3) ppm. HRMS (APCI): $[M+H]^+$ calculated for $C_{11}{}^{13}CH_{21}NO_5H^+$ *m*/*z* 261.1526; found *m*/*z* 261.1523. Optical rotation: $[\alpha]_D^{25}$ = –16.8 (*c* 0.22, MeOH).

Following the same procedure as for (*S*)-**10**, (*R*)-**9** (50 mg, 0.14 mmol) was converted into (*R*)-**10** that was obtained as a white solid (32 mg, 0.12 mmol, 86%). Optical rotation: $[\alpha]_D^{25}$ = +16.0 (*c* 0.15, MeOH).

Synthesis of S-(2-acetamidoethyl) (*S*)- and (*R*)-4-(2-hydroxy-4-methylpentanamido)-(1-^{13}C)butanethioate ((*S*)-11 and (*R*)-11

To a solution of (*S*)-**10** (23 mg, 0.09 mmol) in methanol (1 mL) and H_2O (1 mL), Et_3N (61 mg, 84 µL, 0.60 mmol) was added and the mixture was stirred for 1 h. The mixture was concentrated and the residue was dissolved in CH_2Cl_2 (6 mL), DMAP (6 mg, 0.05 mmol), EDC·HCl (25 mg, 0.13 mmol) and *N*-acetylcysteamine (17 mg, 15 µL, 0.14 mmol) were added to this solution. The mixture was stirred overnight at room temperature and then concentrated under reduced pressure. The residue was purified by column chromatography on silica gel (EtOAc) to yield (*S*)-**11** (19 mg, 0.06 mmol, 67%) as a colourless solid. ^{1}H NMR (700 MHz, MeOD): δ_H 4.02 (dd, $^3J_{H,H}$ = 9.7 Hz, $^3J_{H,H}$ = 3.6 Hz, 1H), 3.33 (t, $^3J_{H,H}$ = 6.6 Hz, 2H), 3.24 (t, $^3J_{H,H}$ = 6.9 Hz, 2H), 3.01 (td, $^3J_{H,H}$ = 6.6 Hz, $^3J_{H,H}$ = 4.6 Hz, 2H), 2.62 (td, $^3J_{H,H}$ = 7.4 Hz, $^3J_{H,H}$ = 5.9 Hz, 2H), 1.95 (s,

3H), 1.88–1.82 (m, 3H), 1.54 (ddd, $^2J_{H,H}$ = 13.8 Hz, $^3J_{H,H}$ = 9.2 Hz, $^3J_{H,H}$ = 3.7 Hz, 1H), 1.47 (ddd, $^2J_{H,H}$ = 13.8 Hz, $^3J_{H,H}$ = 9.7 Hz, $^3J_{H,H}$ = 4.8 Hz, 1H), 0.95 (d, $^3J_{H,H}$ = 6.6 Hz, 6H) ppm; ^{13}C NMR (176 MHz, MeOD): δ_C 200.0 ($^{13}C_q$), 178.1 (C_q), 173.5 (C_q), 71.4 (CH), 44.9 (CH_2), 42.0 (d, $^1J_{C,C}$ = 46.0 Hz, CH_2), 40.1 (CH_2), 39.0 (d, $^3J_{C,C}$ = 4.0 Hz, CH_2), 29.3 (d, $^2J_{C,C}$ = 1.1 Hz, CH_2), 26.4 (d, $^2J_{C,C}$ = 2.2 Hz, CH_2), 25.6 (CH), 23.9 (CH_3), 22.5 (CH_3), 21.8 (CH_3) ppm. NMR data are in agreement with those published previously for the unlabelled compound [9]. HRMS (APCI): $[M+H]^+$ calculated for $C_{13}{}^{13}CH_{26}N_2O_4SH^+$ *m*/*z* 320.1720; found *m*/*z* 320.1716. Optical rotation: $[\alpha]_D^{25}$ = –24.0 (*c* 0.05, MeOH).

Following the same procedure as for (*S*)-**11**, (*R*)-**10** (30 mg, 0.12 mmol) was converted into (*R*)-**11** (22 mg, 0.07 mmol, 60%) was obtained as a colourless solid. Optical rotation: $[\alpha]_D^{25}$ = +22.5 (*c* 0.12, MeOH).

BaeJ-KS2 activity assays

The acylation reactions were performed in incubation buffer (25 mM Tris, 0.5 M NaCl, 10% (v/v) glycerol, pH 7.6) [2]. A solution of enzyme (BaeJ-KS2 and BaeJ-KS2-C222A) in incubation buffer (500 µL, enzyme concentration adjusted to 100 µM) was added to the ^{13}C-labelled SNAC esters (*S*)- or (*R*)-**11** (1 mg dissolved in 5 µL DMSO). After incubation for 30 minutes at 25 °C, the incubation buffer of the reaction mixture potentially containing the free substrate surrogates was exchanged through repeated centrifugation using an ultrafiltration centrifugal tube (3 kDa cut-off), followed by the addition of incubation buffer. Through this method, 5 sequential 10-fold dilutions were achieved. The protein solution was analysed by ^{13}C NMR spectroscopy (Figures 1B and 1F). The filtrates obtained from the first and the fifth centrifugation were also collected and analysed by ^{13}C NMR spectroscopy, showing the presence of free **11** after the first centrifugation step (Figure 1C and 1G), but not after the last round of centrifugation (Figure 1D). In a control experiment, free **11** dissolved in incubation buffer was incubated at 25 °C for 30 minutes, followed by ^{13}C-NMR analysis (Figure 1A).

After the five-fold buffer exchange described above, the BaeJ-KS2 protein samples were lysed using proteinase K (50 mg/mL, working concentration 1 mg/mL) at 55 °C for 30 minutes, followed again by a buffer exchange through centrifugation using an ultrafiltration centrifugal tube (3 kDa cut-off) and analysis of the filtrate by ^{13}C NMR spectroscopy (Figure 1E).

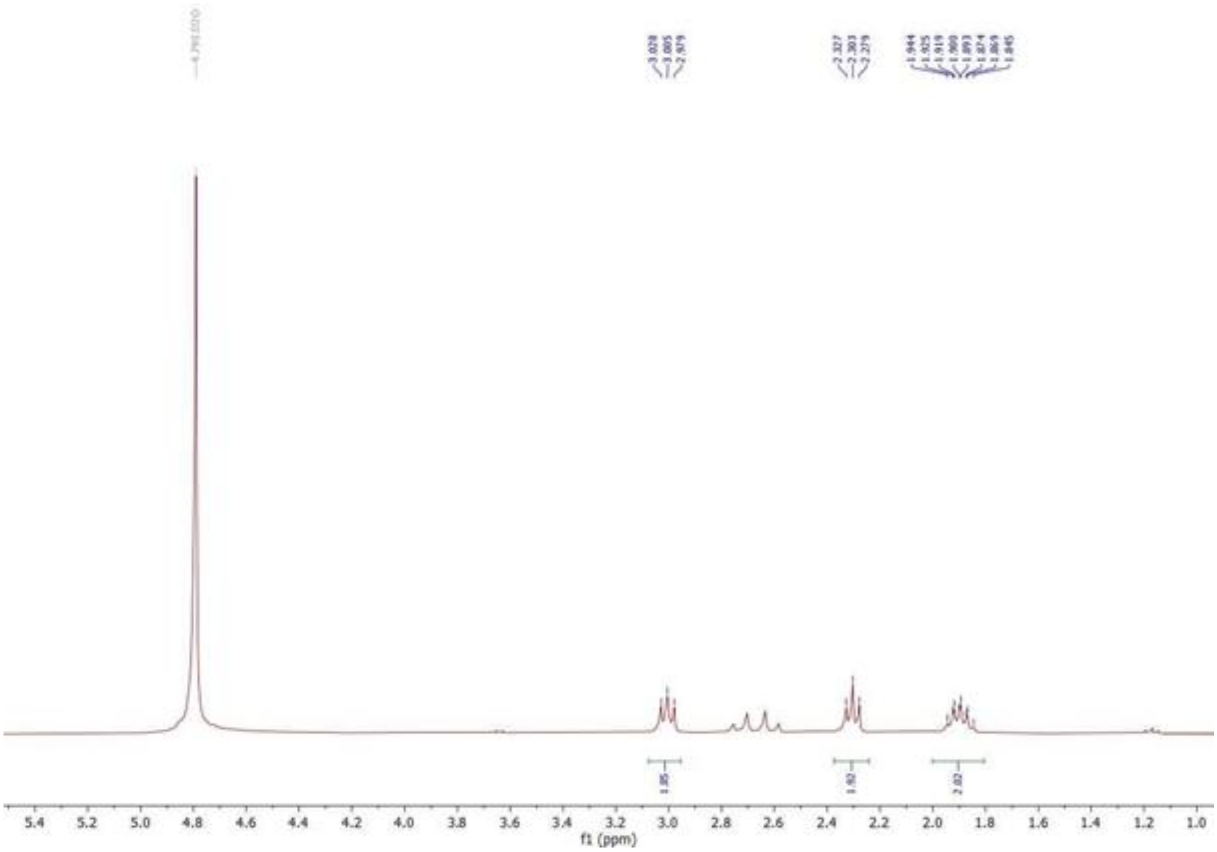

Figure S2. ^{1}H NMR (300 MHz, D_2O) of unlabelled **4**. The additional signals at 2.5–2.8 ppm originate from citric acid.

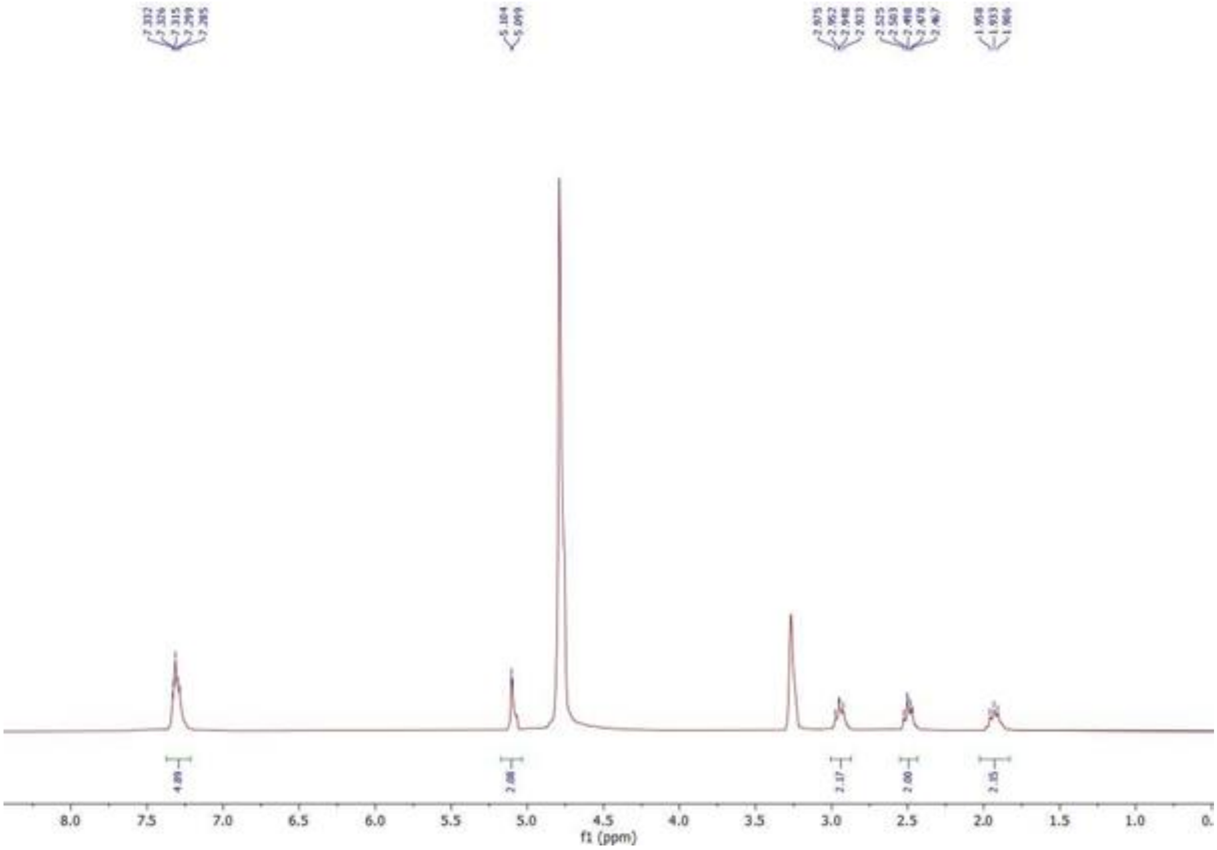

Figure S3. ^{1}H NMR (300 MHz, MeOD) of unlabelled **5**.

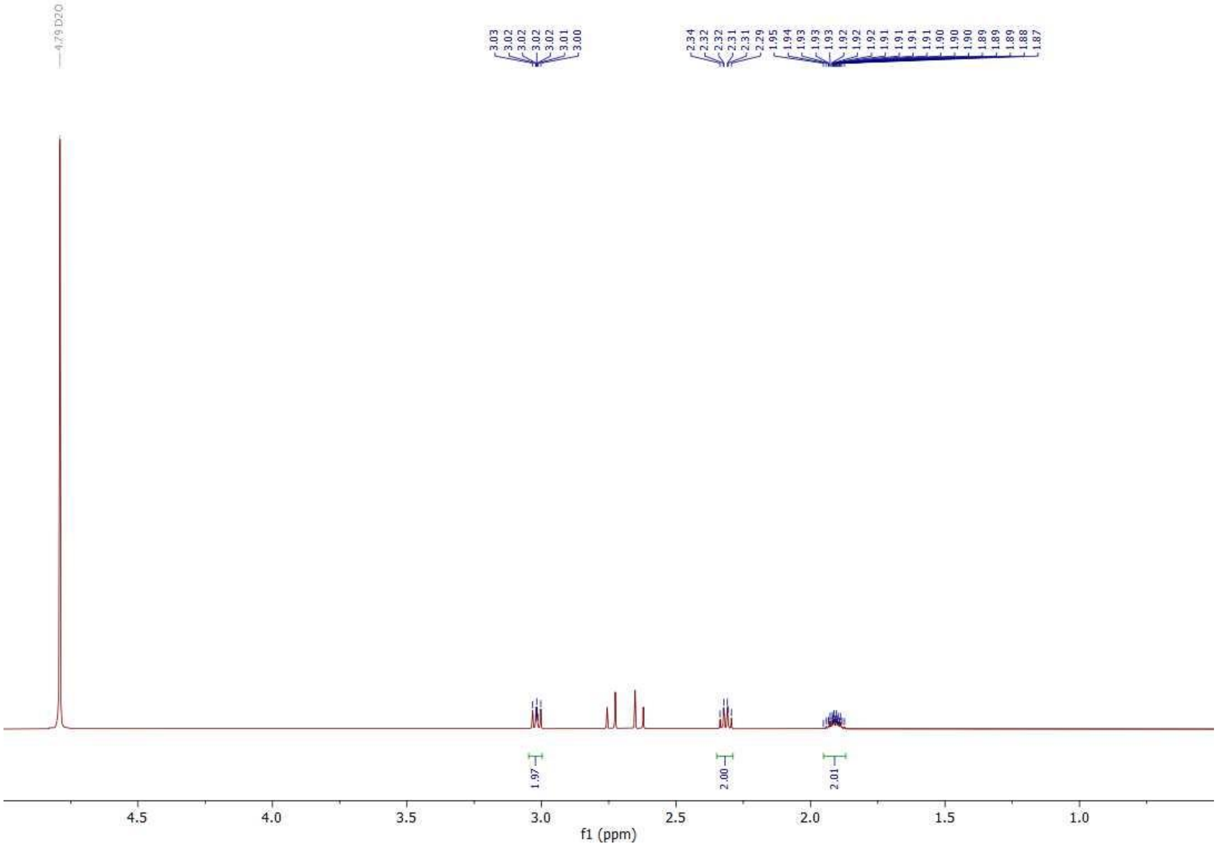

Figure S4. ^{1}H NMR (500 MHz, D_2O) of **4**.

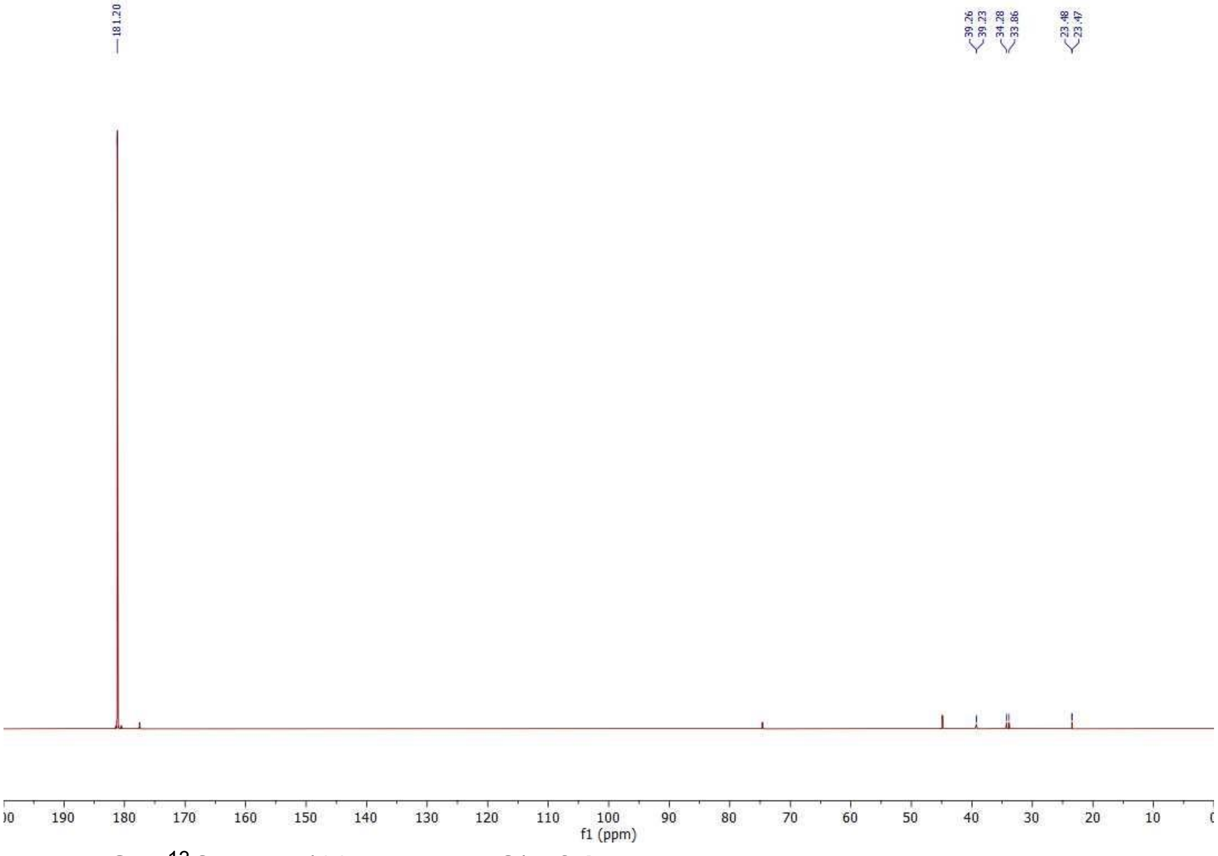

Figure S5. ^{13}C NMR (125 MHz, D_2O) of **4**.

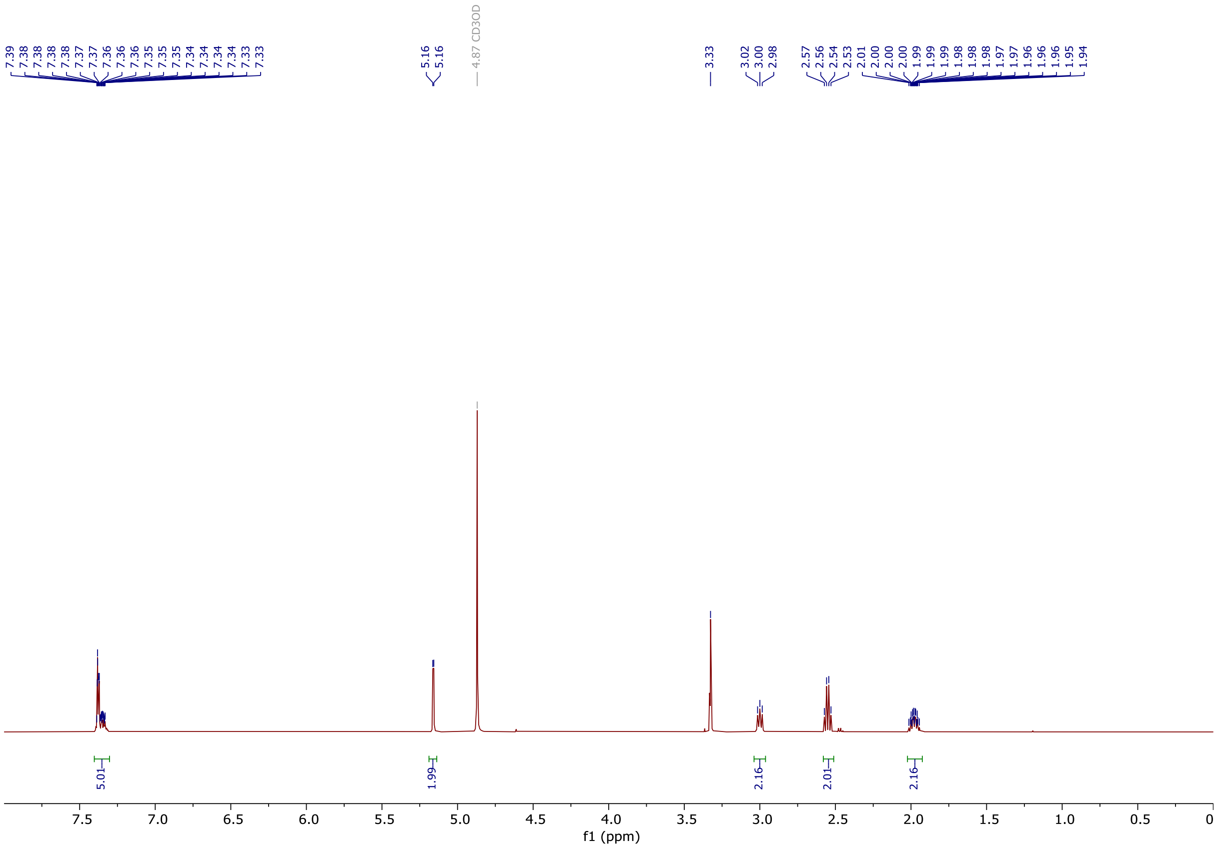

Figure S6. ^{1}H NMR (500 MHz, MeOD) of **5**.

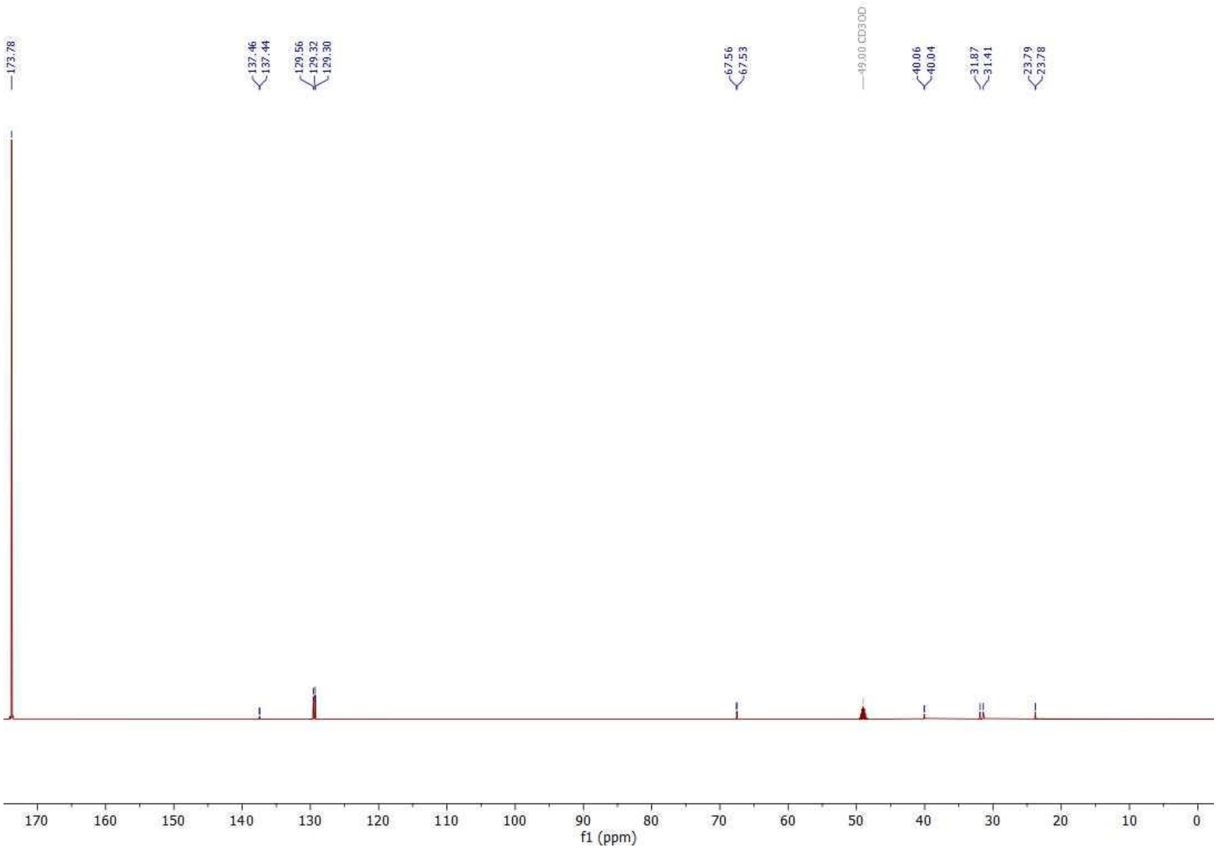

Figure S7. ^{13}C NMR (125 MHz, MeOD) of **5**.

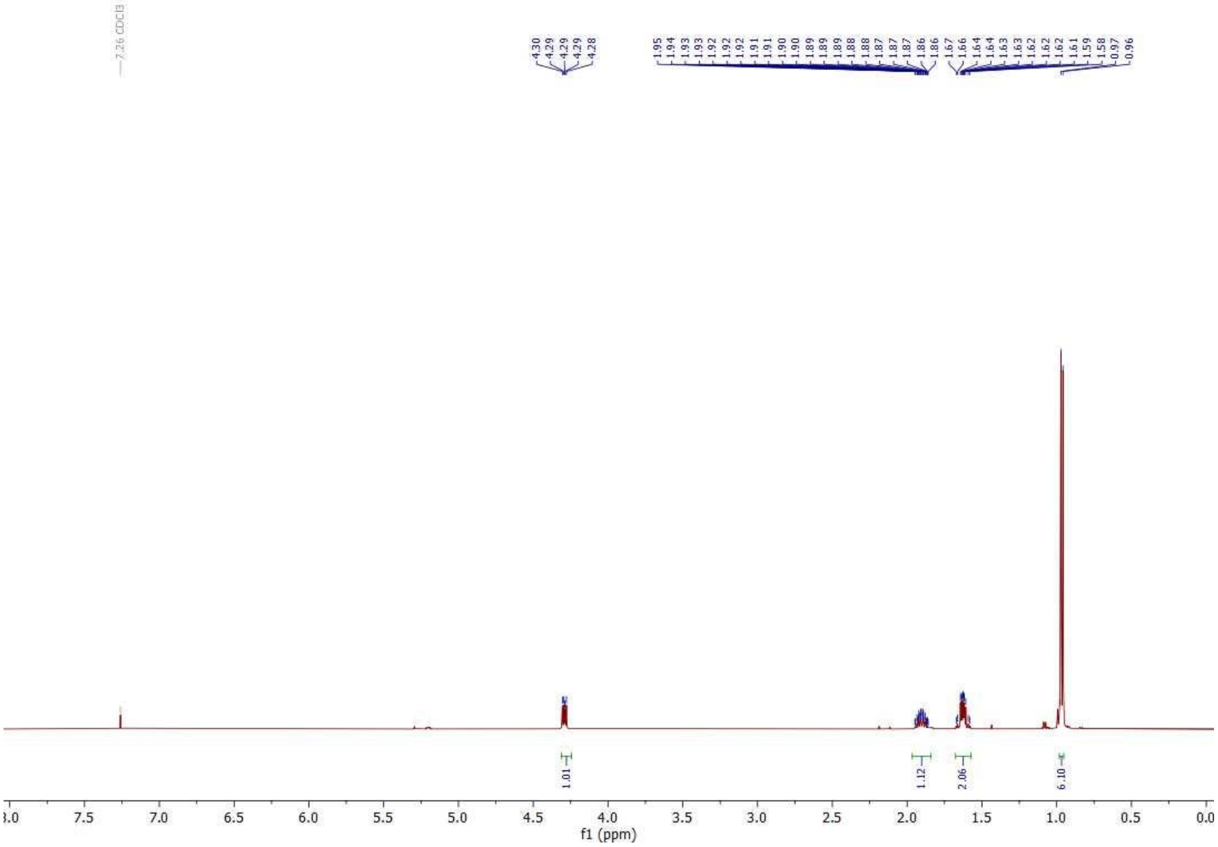

Figure S8. ^{1}H NMR (500 MHz, $CDCl_3$) of **7**.

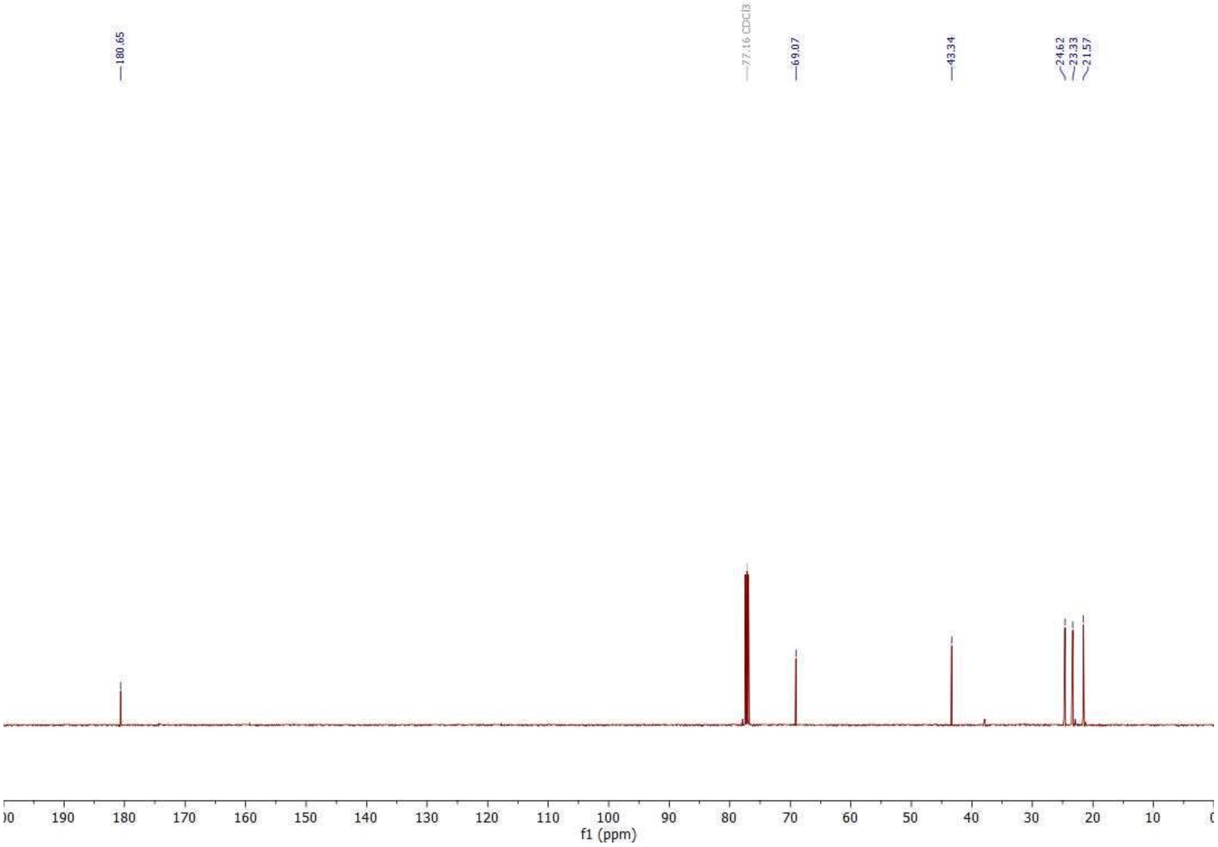

Figure S9. ^{13}C NMR (125 MHz, $CDCl_3$) of **7**.

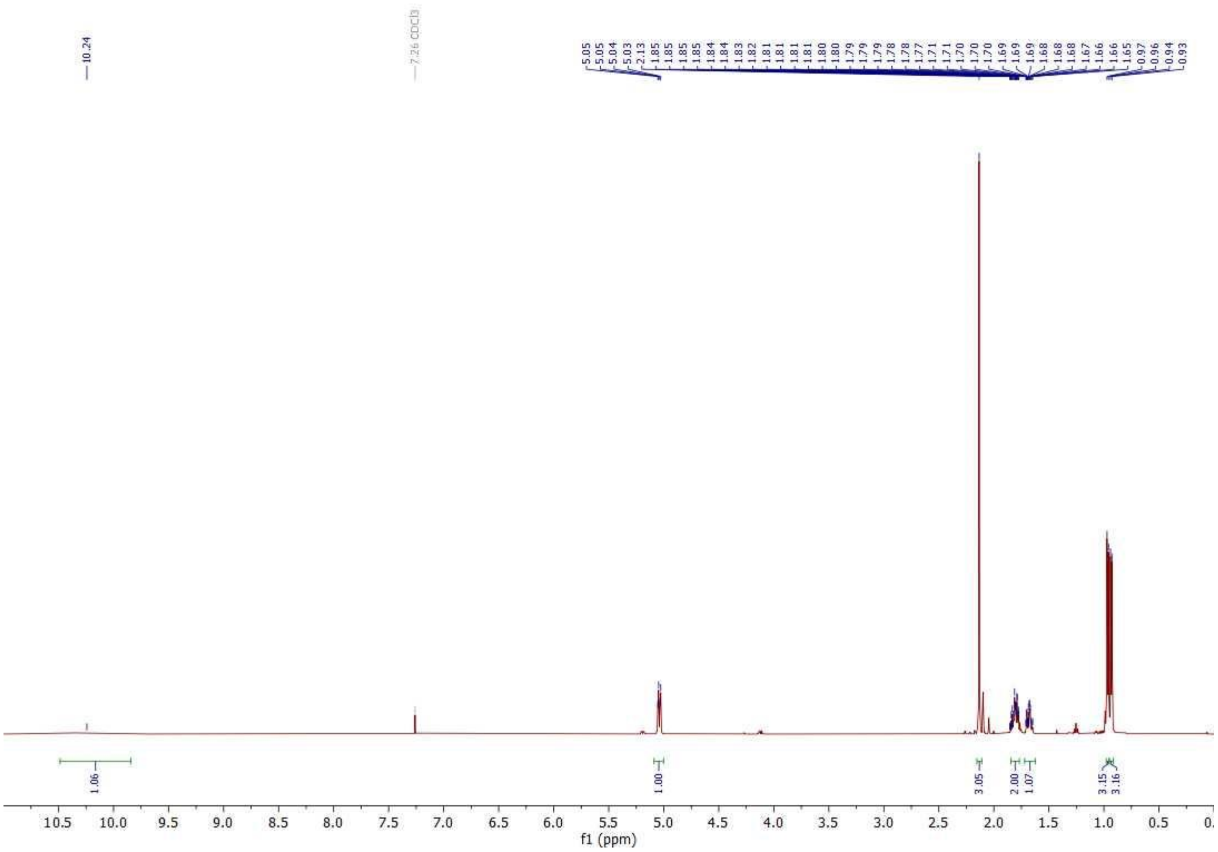

Figure S10. ^{1}H NMR (500 MHz, $CDCl_3$) of **8**.

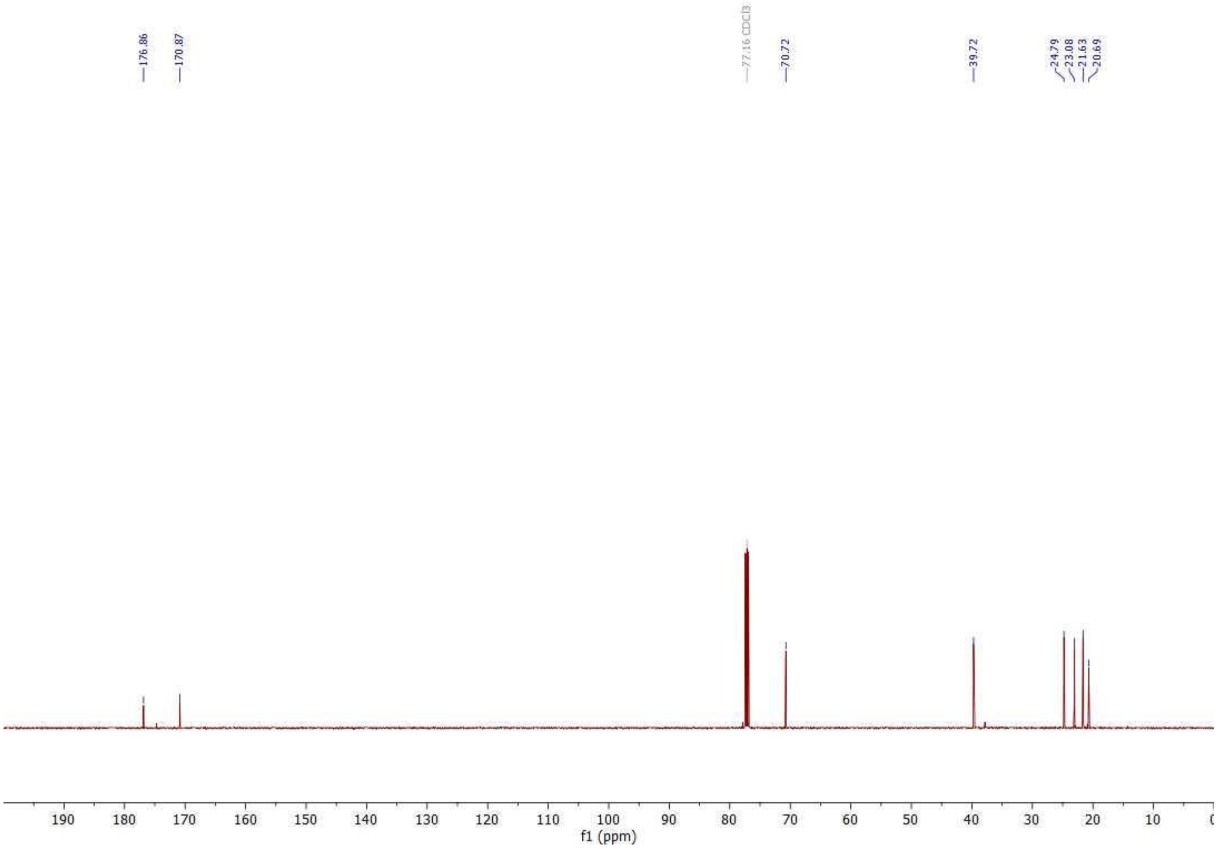

Figure S11. ^{13}C NMR (125 MHz, $CDCl_3$) of **8**.

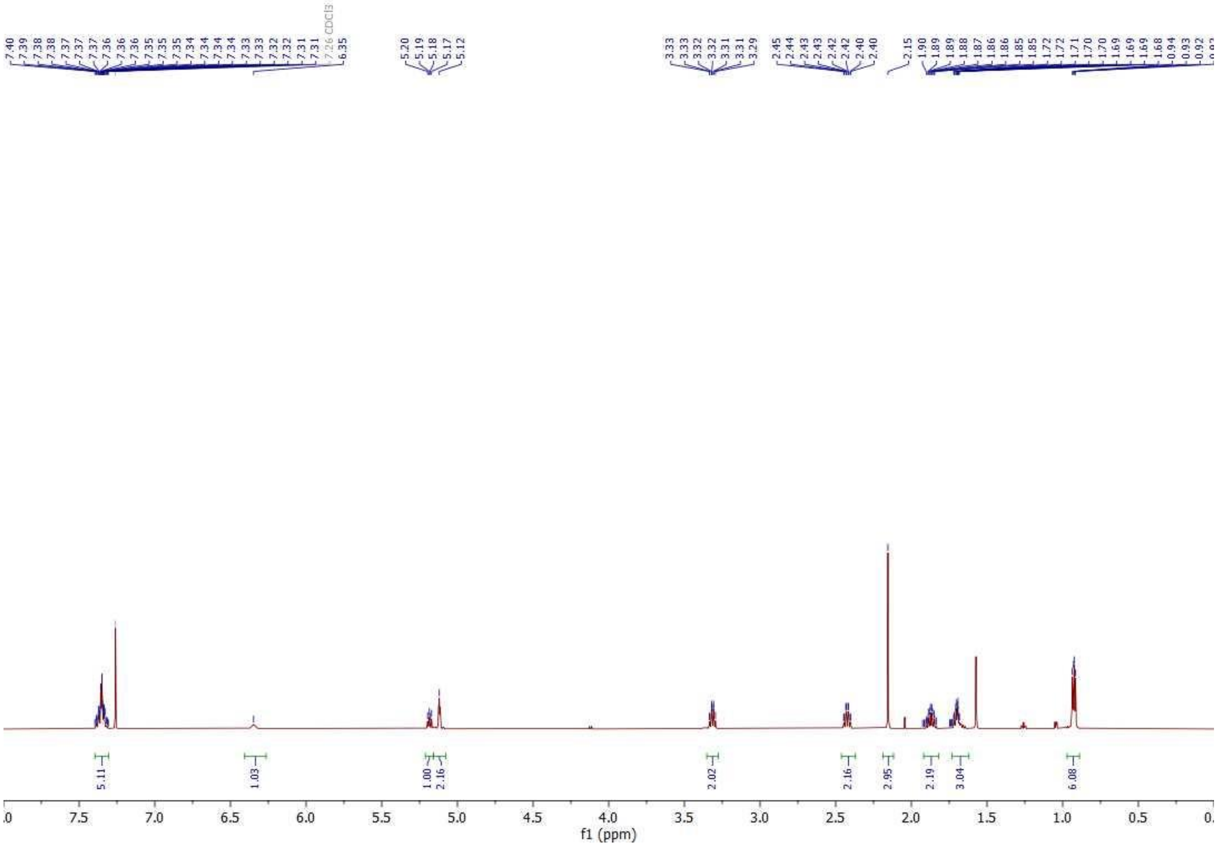

Figure S12. ^{1}H NMR (500 MHz, $CDCl_3$) of **9**.

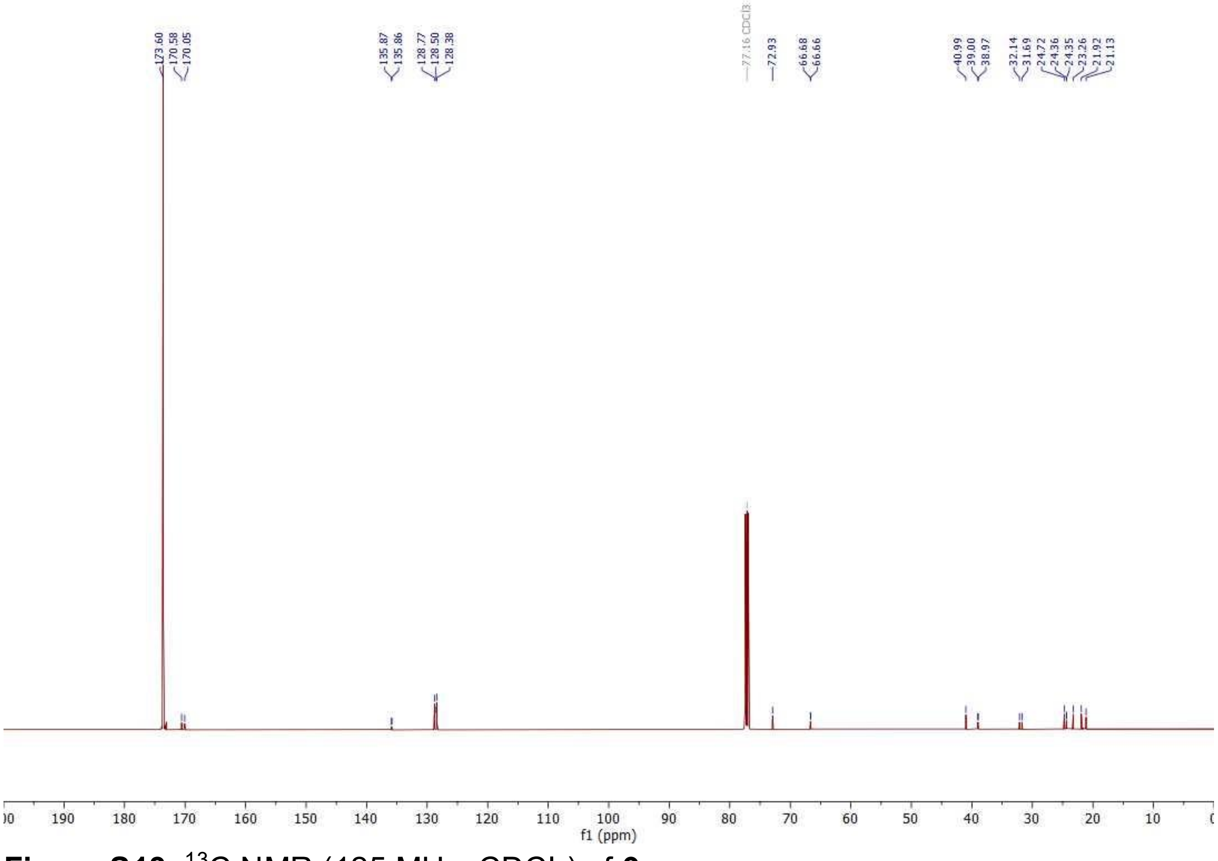

Figure S13. ^{13}C NMR (125 MHz, $CDCl_3$) of **9**.

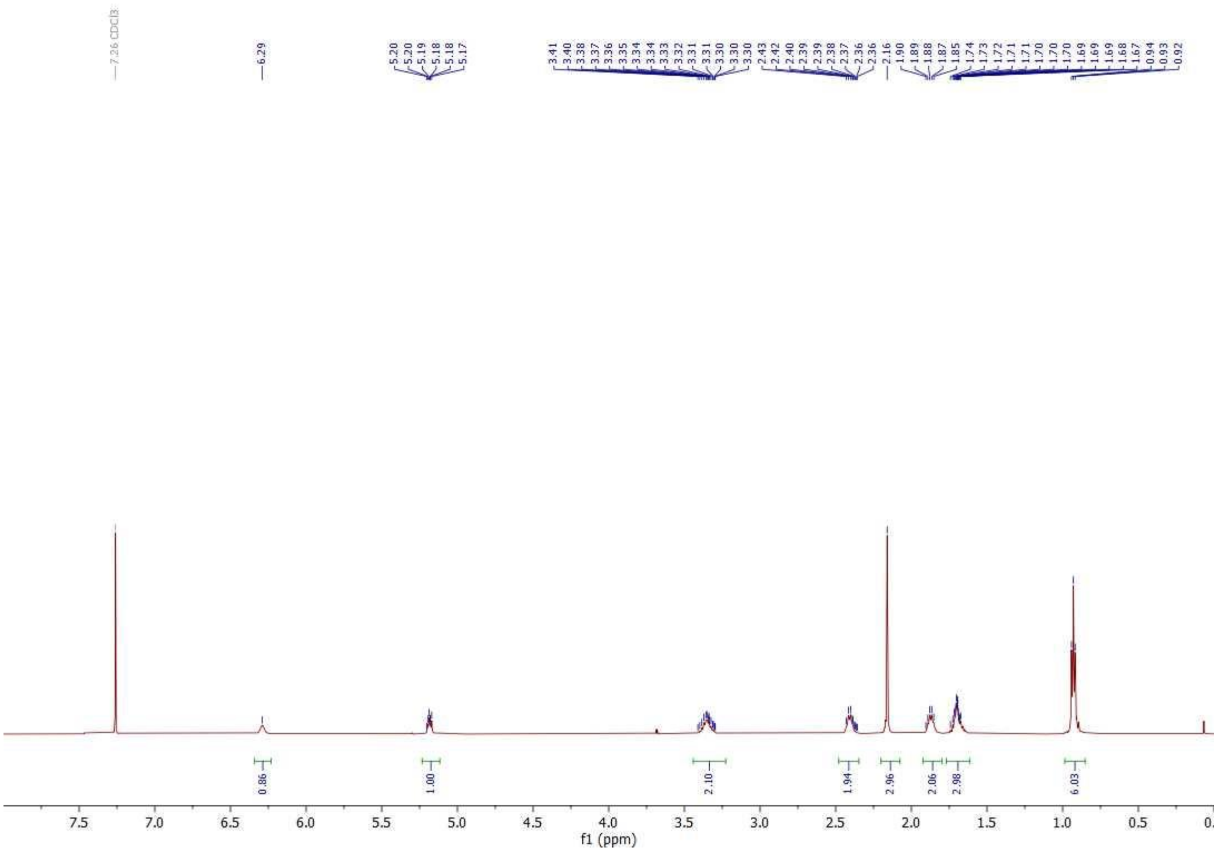

Figure S14. ^{1}H NMR (500 MHz, $CDCl_3$) of **10**.

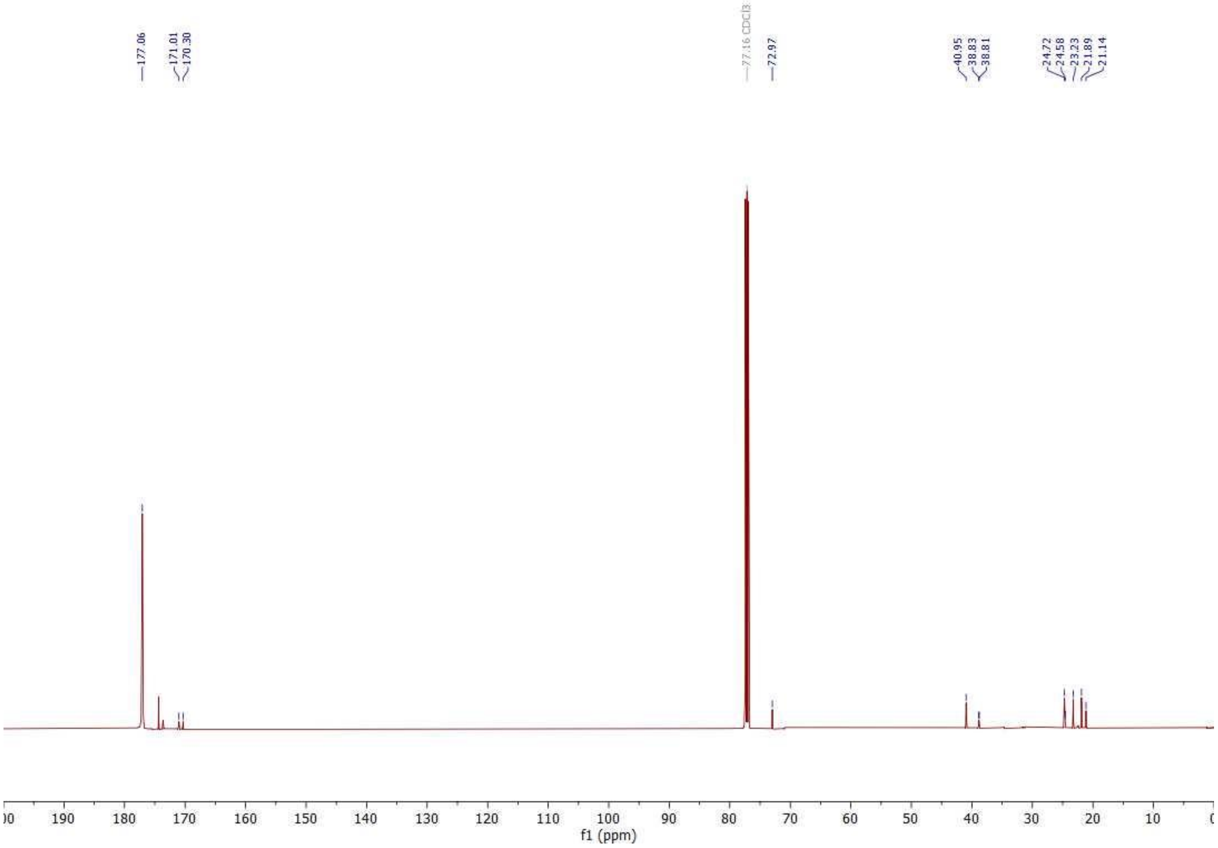

Figure S15. ^{13}C NMR (125 MHz, $CDCl_3$) of **10**.

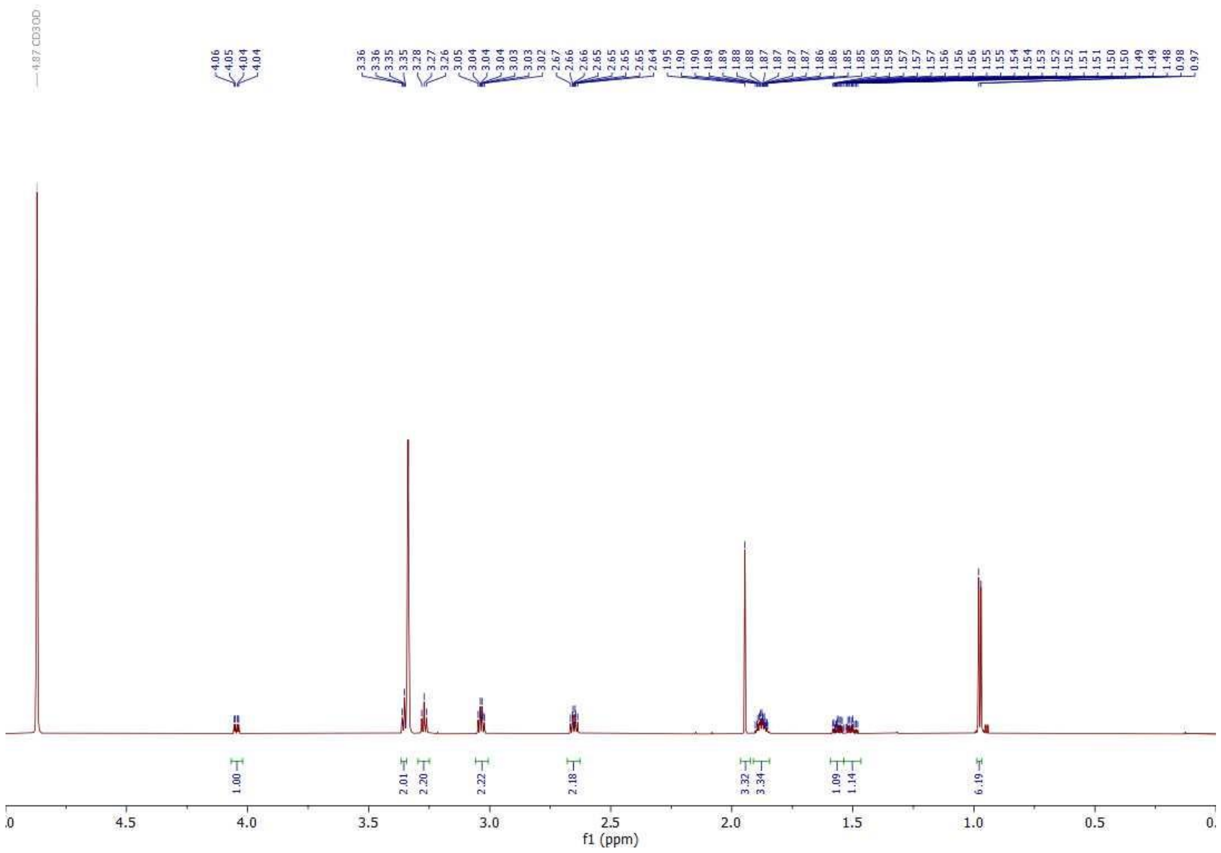

Figure S16. ^{1}H NMR (700 MHz, MeOD) of **11**.

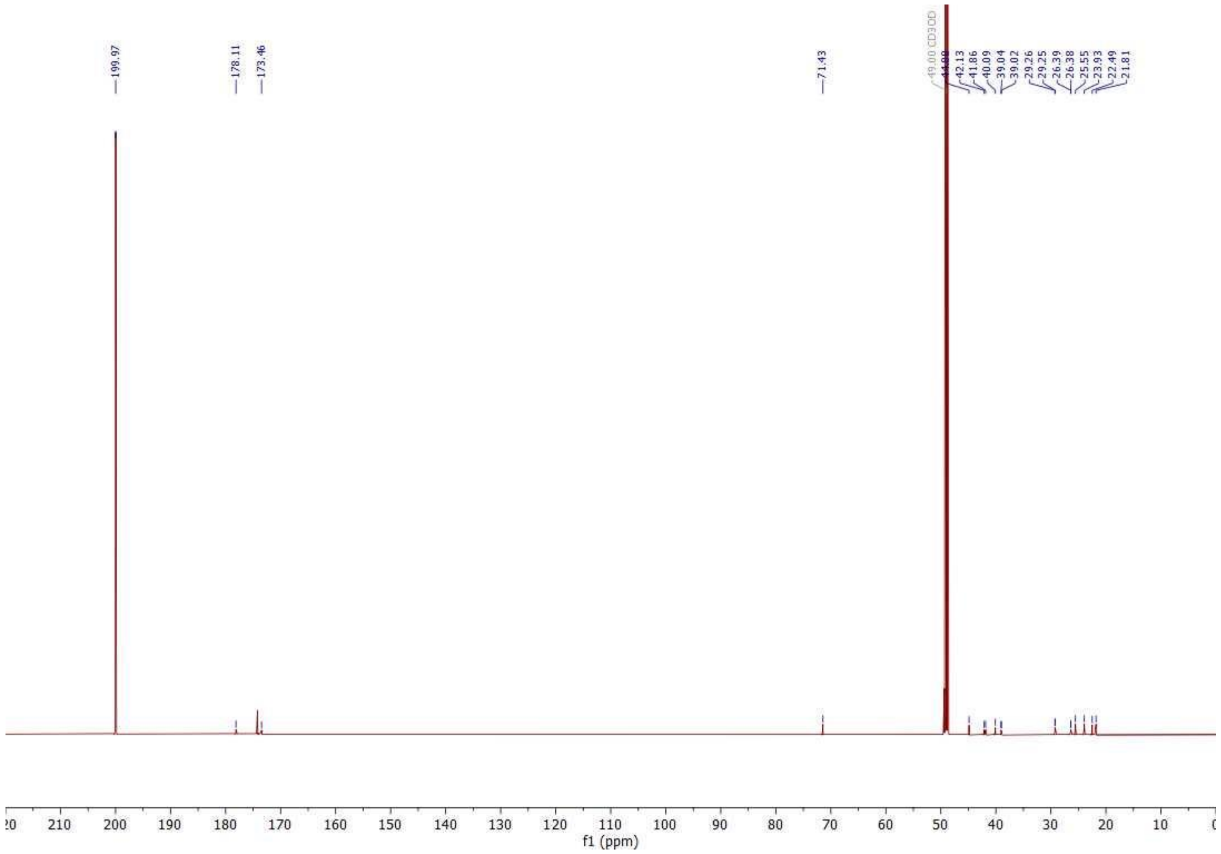

Figure S17. ^{13}C NMR (176 MHz, MeOD) of **11**.

References

1. Smith, D. K.; Kassam, T.; Singh, B.; Elliott, J. F. *J. Bacteriol.,* **1992,** *174,* 5820–5826.

2. Gay, D. C.; Gay, G.; Axelrod, A. J.; Jenner, M.; Kohlhaas, C.; Kampa, A.; Oldham, N. J.; Piel, J.; Keatinge-Clay, A. T. *Structure,* **2014,** *22,* 444–451.

3. Dickschat, J. S.; Pahirulzaman, K. A. K.; Rabe, P.; Klapschinski, T. A. *ChemBioChem,* **2014,** *15,* 810–814.

4. Huang, Y.; Su. L.; Wu, J. *PLoS One,* **2016,***11,* e0157466.

5. Bradford, M. M. *Anal. Biochem.,* **1976,** *72,* 248–254.

6. Kimura, H.; Sampei, S.; Matsuoka, D.; Harada, N.; Watanabe, H.; Arimitsu, K.; Ono, M.; Saji, H. *Bioorg. Med. Chem.,* **2016,** *24,* 2251–2256.

7. Busto, E.; Richter, N.; Grischek B.; Kroutil, W. *Chem. Eur. J.,* **2014,** *20,* 11225–11228.

8. Kolasa, H. T.; Miller, M. J. *J. Org. Chem.,* **1987,** *52,* 4978–4984.

9. Piasecki, S. K.; Zheng, J.; Axelrod, A. J.; Detelich, M. E.; Keatinge-Clay, A. T. *Proteins: Struct. Funct.,* **2014,** *82,* 2067–2077.
